# Late-onset Cognitive Impairment and Modifiable Risk Factors in Adult Childhood Cancer Survivors

**DOI:** 10.1001/jamanetworkopen.2023.16077

**Published:** 2023-05-31

**Authors:** Nicholas S. Phillips, Kayla L. Stratton, AnnaLynn M. Williams, Tim Ahles, Kirsten K. Ness, Harvey Jay Cohen, Kim Edelstein, Yutaka Yasui, Kevin Oeffinger, Eric J. Chow, Rebecca M. Howell, Leslie L. Robison, Gregory T. Armstrong, Wendy M. Leisenring, Kevin R. Krull

**Affiliations:** 1 Epidemiology and Cancer Control Department, St Jude Children’s Research Hospital, Memphis, Tennessee; 2 Department of Public Health Sciences, Fred Hutchinson Cancer Center, Seattle, Washington; 3 Department of Psychiatry and Behavioral Sciences, Memorial Sloan Kettering Cancer Center, New York City, New York; 4 Department of Medicine, Duke University School of Medicine, Durham, North Carolina; 5 Department of Psychology, Princess Margaret Cancer Centre, Toronto, Ontario, Canada; 6 Department of Radiation Physics, MD Anderson Cancer Center, Houston, Texas

## Abstract

**Importance:**

Long-term survivors of childhood cancer may be at elevated risk for new neurocognitive impairment and decline as they age into adulthood.

**Objective:**

To determine whether aging adult childhood cancer survivors report more new-onset neurocognitive impairments compared with their siblings and to identify risk factors associated with such impairments.

**Design, Setting, and Participants:**

Participants of this cohort study included adult survivors of childhood cancer from the Childhood Cancer Survivor Study and their siblings as a control group. The original cohort included survivors who received a diagnosis between January 1, 1970, and December 31, 1986, for whom longitudinal neurocognitive assessment was available. This study examined the prevalence of new-onset neurocognitive impairment between baseline (23.4 years after diagnosis) and follow-up (35.0 years after diagnosis). The analysis was performed from January 2021 to May 2022.

**Exposures:**

Cancer treatment exposures were abstracted from medical records. Chronic health conditions were graded using Common Terminology Criteria for Adverse Events version 4.03.

**Main Outcomes and Measures:**

The primary outcome was new-onset (present at follow-up, but not present at baseline) neurocognitive impairment (defined as a score in the worst 10% of the sibling cohort). Impairment was assessed using the Childhood Cancer Survivor Study Neurocognitive questionnaire. Relative risks (RRs) and 95% CIs were used to estimate associations of neurocognitive impairment with treatment and health behaviors and conditions using generalized linear models.

**Results:**

The cohort comprised 2375 survivors (mean [SD] age at evaluation, 31.8 [7.5] years; 1298 women [54.6%]) of childhood cancer, including acute lymphoblastic leukemia (ALL; 1316 participants), central nervous system (CNS) tumors (488 participants), and Hodgkin lymphoma (HL; 571 participants). A total of 232 siblings (mean [SD] age at evaluation, 34.2 [8.4] years; 134 women [57.8%]) were included. Compared with siblings, a higher proportion of survivors with no impairment in memory at baseline had new-onset memory impairment at follow-up: siblings proportion, 7.8% (95% CI, 4.3%-11.4%); ALL survivors treated with chemotherapy only, 14.0% (95% CI, 10.7%-17.4%); ALL survivors treated with cranial radiation (CRT), 25.8% (95% CI, 22.6%-29.0%); CNS tumor survivors, 34.7% (95% CI, 30.0%-39.5%); and HL survivors, 16.6% (95% CI, 13.4%-19.8%). New-onset memory impairment was associated with CRT in CNS tumor survivors (RR, 1.97; 95% CI, 1.33-2.90) and alkylator chemotherapy greater than or equal to 8000 mg/m^2^ in ALL survivors treated without CRT (RR, 2.80; 95% CI, 1.28-6.12). Neurologic conditions mediated the impact of CRT on new-onset memory impairment in CNS survivors. Smoking, low educational attainment, and low physical activity were associated with elevated risk for new-onset memory impairment.

**Conclusions and Relevance:**

These findings suggest that adult survivors of childhood cancer are at elevated risk for late-onset memory impairment related to modifiable risk factors identified early in survivorship.

## Key Points

**Question:**

Are adult survivors of childhood cancer at risk for new-onset neurocognitive impairments that outpace those associated with typical aging?

**Findings:**

In this cohort study of 2375 adult survivors of childhood cancer and their sibling controls, new-onset memory impairment emerged more often in survivors decades after cancer diagnosis and treatment. The increased risk was associated with cancer treatment, modifiable health behaviors, and chronic health conditions.

**Meaning:**

These findings suggest that adult survivors of childhood cancer are at elevated risk for new-onset neurocognitive impairments as they age and that such new-onset impairment may be an indicator of future neurocognitive decline and possibly dementia.

## Teaser

This cohort study examines whether aging adult childhood cancer survivors report more new-onset neurocognitive impairments compared with their siblings and identifies factors associated with such impairments.

## Introduction

At present, more than 85% of US children who receive a diagnosis of and treatment for cancer will become 5-year survivors, contributing to a growing population of more than 500 000 adult childhood cancer survivors aged 20 to 39 years.^1^ It is well-established that these survivors are at elevated risk for severe or disabling late effects,^2,3,4^ including neurocognitive impairment that emerges 5 to 10 years from diagnosis.^5,6,7^ However, it is unknown whether survivors who do not demonstrate domain-specific neurocognitive impairments (eg, memory) in the first 10 years after therapy remain at elevated risk for new-onset impairment as they age through adulthood. Self-report neurocognitive questionnaires provide an inexpensive, validated, and easy to implement tool to screen for neurocognitive impairment.^8,9^ Previous studies^10,11,12^ in populations without cancer have demonstrated that self-reported neurocognitive impairment is associated with increased risk of future cognitive declines. If new-onset neurocognitive problems are emerging later in adult survivors, self-reported neurocognitive impairments play an important role in screening aging survivors for deficits and dementia risk.^13^

Younger age at diagnosis and receipt of central nervous system (CNS)–directed therapy are associated with increased risk of neurocognitive impairment in young survivors.^14,15,16,17^ Many survivors are also at risk of developing chronic health conditions,^18^ which further increase the risk of impairment.^19,20^ The effects of CNS-directed therapies may alter brain development and reduce neurocognitive reserve needed to compensate for brain stressors and chronic health conditions related to typical aging.^21^ Cardiopulmonary toxic therapies can increase risk for chronic health conditions that impact brain function.^19^ These processes may promote accelerated neurocognitive decline and elevate risk for early onset cognitive impairments, even in survivors with no history of neurocognitive impairment shortly after therapy (ie, a low-risk group) or no history of CNS-directed therapy. The current study aimed to determine whether adult (aged ≥18 years) childhood cancer survivors with no impairment at baseline (after completion of therapy) reported new-onset neurocognitive impairment during subsequent long-term follow-up and, if so, what factors were associated with that decline.

## Methods

### Participants

The Childhood Cancer Survivor Study (CCSS) is a retrospective cohort with longitudinal follow-up of survivors of childhood cancer diagnosed before age 21 years, treated at 1 of 31 institutions in North America, and who survived at least 5 years after diagnosis. The original cohort included survivors who received a diagnosis between January 1, 1970, and December 31, 1986, for whom longitudinal neurocognitive assessment is available. The CCSS methods and study design have previously been described in detail.^22^ The CCSS was approved by the institutional review boards at all participating sites, and participants provided written informed consent. This study followed the Strengthening the Reporting of Observational Studies in Epidemiology (STROBE) reporting guideline for observational studies.

Survivors, and a randomly selected sibling comparison group, were aged 18 years or older at the time they completed a neurocognitive questionnaire at baseline (2003-2004) and subsequent neurocognitive questionnaire at follow-up (2014-2015) (eFigure 1 and eFigure 2 in Supplement 1). Survivors and siblings who were impaired in all neurocognitive domains at baseline and those with a genetic disorder that predisposes to neurocognitive decline not related to cancer (eg, trisomy 21 or Turner syndrome) were excluded from analyses.

Survivors who had 1 of the 3 most common diagnoses were included in the analyses: CNS tumors, Hodgkin lymphoma (HL), and acute lymphoblastic leukemia (ALL). Those with ALL were further divided into those treated with chemotherapy only and those treated with cranial radiation therapy (CRT). These 4 groups were compared with a sibling control group of CCSS survivors to determine differences in prevalence of impairments.

### Exposures and Covariates

Cancer diagnosis and treatment exposures were abstracted from medical records at the treating institution. The maximum CRT target dose was determined for each individual.^23^ Among ALL survivors, methotrexate was assessed by patterns of administration route.^24^ Chronic health conditions were obtained using a 289-item questionnaire assessing diagnoses and symptoms and age at onset^25^ and were graded for severity according to the National Cancer Institute’s Common Terminology Criteria for Adverse Events version 4.03 (scored as 1, mild; 2, moderate; 3, severe; and 4, life-threatening).^4^ For this study, analysis of health conditions was limited to cardiopulmonary, endocrine, and neurologic systems given their recognized association with neurocognitive outcomes.^19^ Chronic health conditions were classified as prevalent at baseline or with new onset by follow-up. Anxiety and depression scores were obtained from the Brief Symptom Inventory–18.^26^ Smoking status, social attainment, and physical activity were collected at baseline. Data on race and ethnicity were obtained from self-report questionnaires and are included in CCSS studies to assess health disparities and inequities in participation and outcomes.

### Neurocognitive Outcomes

Survivors and siblings completed the CCSS Neurocognitive Questionnaire (NCQ), a validated rating scale (ranging from 1, never a problem, to 3, often a problem) developed for the assessment of neurocognitive problems in adult survivors.^8^ The 32-item CCSS-NCQ^25^ used items representing the cognitive constructs of organization, memory, initiation and processing speed, and emotional regulation. The CCSS-NCQ groups along these 4 factors and has been validated against objective testing.^9^ A binary outcome of impairment in each domain was defined as a score in the worst 10% of the CCSS sibling cohort on the basis of the distribution of that domain score (mean and SD) of all siblings tested at each survey. In addition to the siblings who completed the baseline and follow-up, an additional 1885 siblings were offered and completed the follow-up questionnaire. Their data are not included in this analysis but were used to establish norms.^27^

### Statistical Analysis

For each of the 4 diagnosis groups (ALL with chemotherapy only, ALL with CRT, HL, and CNS tumor), generalized linear models with log link, Poisson errors, and robust variances were used to determine relative risk (RR) of new-onset impairment for each neurocognitive domain at follow-up, among survivors who did not report impairment in that domain at baseline. Impairment of survivors at follow-up was compared with that of siblings using the aforementioned model structure. Using stepwise selection, a best fit model was determined for treatment factors, for survivors only, within each diagnosis group, starting with treatments relevant for the diagnosis, with a *P* < .05 for retention in the model. Similar models were generated using factors associated with chronic health problems instead of treatment and separately using health behaviors. All models were adjusted for age and sex. Survivors with missing treatment data were not included in the multivariable treatment models. Chronic health conditions and health behaviors found to be significantly associated with impairment were further tested as mediators of treatment effects via additional generalized structural equation models. Estimates of indirect effects and proportion mediated (PM) were reported. All *P* values reported are 2-sided and are considered significant at the *P* < .05 level, although because of the number of statistical tests performed, some caution should be taken when viewing *P* values between .01 and .05. Analyses were performed using SAS statistical software version 9.4 (SAS Institute), R statistical software version 3.5.2 (R Project for Statistical Computing), and Stata/SE statistical software version 17.0 (StataCorp). The analysis was conducted from January 2021 to May 2022.

## Results

**Figure 1.**
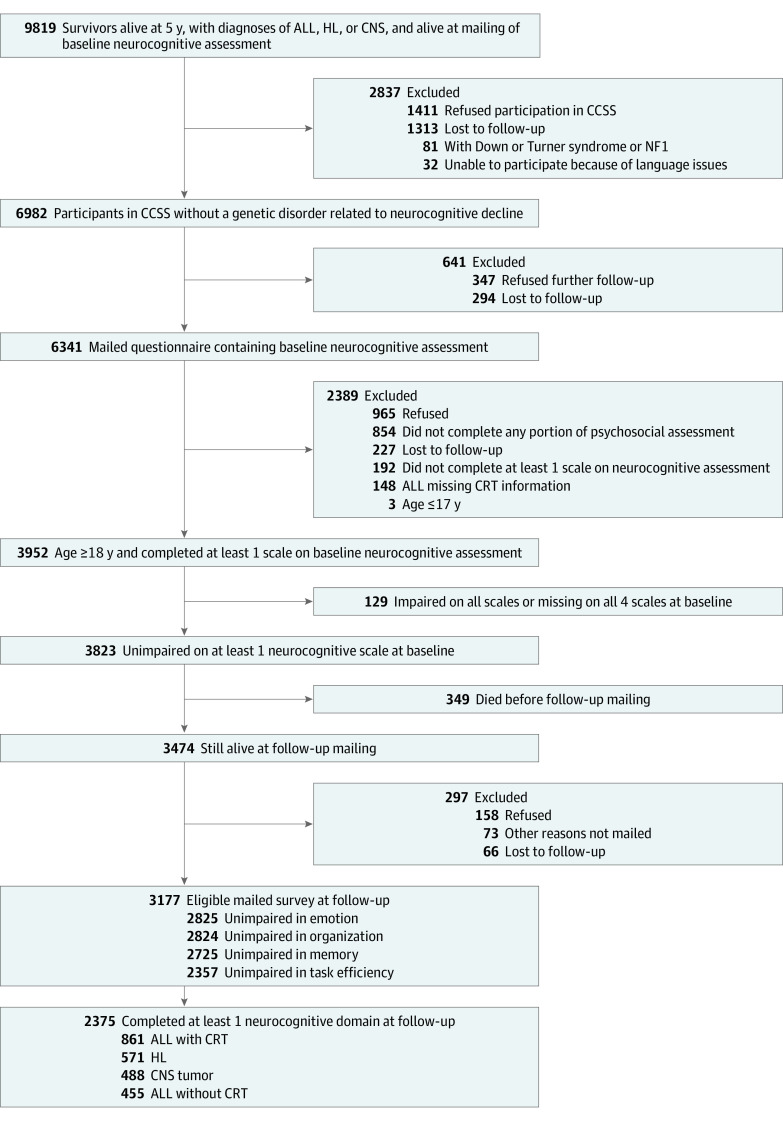
Participant Enrollment Flowchart ALL indicates acute lymphoblastic leukemia; CCSS, Childhood Cancer Survivor Study; CNS, central nervous system; CRT, cranial radiation therapy; HL, Hodgkin lymphoma; NF1, neurofibromatosis type 1.

Treatment data are included in eTable 1 in Supplement 1, and a detailed description of incidence of chronic conditions is shown in eTable 2 in Supplement 1. Sociodemographic and behavioral data are presented in Table 1. Of the potentially eligible 3952 5-year childhood cancer survivors with a diagnosis of ALL, HL, or CNS tumor, 3823 (97%) did not report impairment on at least 1 neurocognitive scale at baseline (Figure 1 and eFigures 1 and 2 in Supplement 1). At follow-up, 3474 participants (91%) were alive, of whom 2375 (68%) (mean [SD] age at evaluation, 31.8 [7.5] years; 1298 women [54.6%]) completed at least 1 neurocognitive domain. Cancers experienced during childhood included ALL (1316 participants; 455 treated with chemotherapy only and 861 treated with CRT), CNS tumors (488 participants), and HL (571 participants). The mean (SD) interval between baseline and follow-up was 11.6 (0.7) years (baseline, 23.4 years after diagnosis; follow-up, 35.0 years after diagnosis). A total of 232 siblings (mean [SD] age at evaluation, 34.2 [8.4] years; 134 women [57.8%]) were included for comparison.

**Table 1.** Characteristics of Survivors of Childhood Cancer Overall and by Diagnosis Group Compared With Siblings at Baseline

Characteristic	Participants, No. (%)				
	CNS tumors (n = 488)	HL (n = 571)	ALL without CRT (n = 455)	ALL with CRT (n = 861)	Siblings (n = 232)
Sex					
Female	255 (52.3)	313 (54.8)	270 (59.3)	460 (53.4)	134 (57.8)
Male	233 (47.7)	258 (45.2)	185 (40.7)	401 (46.6)	98 (42.2)
Age at diagnosis, y					
0-4	155 (31.8)	9 (1.6)	269 (59.1)	385 (44.7)	NA
5-9	143 (29.3)	59 (10.3)	123 (27.0)	241 (28.0)	NA
10-14	140 (28.7)	216 (37.8)	43 (9.5)	162 (18.8)	NA
15-20	50 (10.2)	287 (50.3)	20 (4.4)	73 (8.5)	NA
Mean (SD)	7.9 (5.1)	14.2 (3.9)	5.2 (3.9)	6.6 (4.7)	NA
Age at follow-up, y					
18-29	219 (44.9)	34 (6.0)	307 (67.5)	390 (45.3)	76 (32.8)
30-39	209 (42.8)	293 (51.3)	125 (27.5)	396 (46.0)	88 (37.9)
≥40	60 (12.3)	244 (42.7)	23 (5.1)	75 (8.7)	68 (29.3)
Mean (SD)	30.8 (7.1)	38.3 (6.0)	27.1 (6.4)	30.5 (6.2)	34.2 (8.4)
Race					
Non-Hispanic White	462 (95.1)	536 (94.2)	411 (90.7)	792 (92.6)	210 (95.5)
Non-Hispanic other race^a^	24 (4.9)	33 (5.8)	42 (9.3)	63 (7.4)	10 (4.5)
Education					
High school graduate or less	106 (21.7)	61 (10.7)	43 (9.5)	146 (17.0)	29 (12.5)
Some college	161 (33.0)	152 (26.6)	188 (41.3)	306 (35.5)	57 (24.6)
College graduate or higher	221 (45.3)	358 (62.7)	224 (49.2)	409 (47.5)	146 (62.9)
Smoking status					
Current smoker	47 (9.6)	64 (11.2)	57 (12.5)	96 (11.2)	36 (15.5)
Former smoker	55 (11.3)	137 (24.0)	79 (17.4)	90 (10.5)	53 (22.8)
Never smoked	386 (79.1)	370 (64.8)	319 (70.1)	674 (78.4)	143 (61.6)
Body mass index^b^					
<25 (Underweight or healthy)	214 (46.4)	290 (51.7)	258 (58.1)	294 (36.0)	111 (50.0)
25-29 (Overweight)	146 (31.7)	178 (31.7)	121 (27.3)	270 (33.0)	65 (29.3)
≥30 (Obese)	101 (21.9)	93 (16.6)	65 (14.6)	253 (31.0)	46 (20.7)
Met Centers for Disease Control and Prevention activity criteria^c^	270 (56.8)	359 (63.9)	290 (64.7)	480 (57.3)	140 (61.4)

Abbreviations: ALL, acute lymphoblastic leukemia; CNS, central nervous system; CRT, cranial radiation therapy; HL, Hodgkin lymphoma; NA, not applicable.

^a^ Includes individuals who self-reported American Indian/Alaska Native, Asian or Pacific Islander, Black, multiracial, or any other race who did not report being of Hispanic ethnicity.

^b^ Body mass index is calculated as weight in kilograms divided by height in meters squared.

^c^ The Centers for Disease Control and Prevention activity criteria includes at least 150 minutes of moderate-intensity aerobic physical activity or 75 minutes of vigorous-intensity physical activity, or an equivalent combination each week. eTable 2 in Supplement 1 includes detailed description of incidence of chronic conditions.

**Figure 2.**
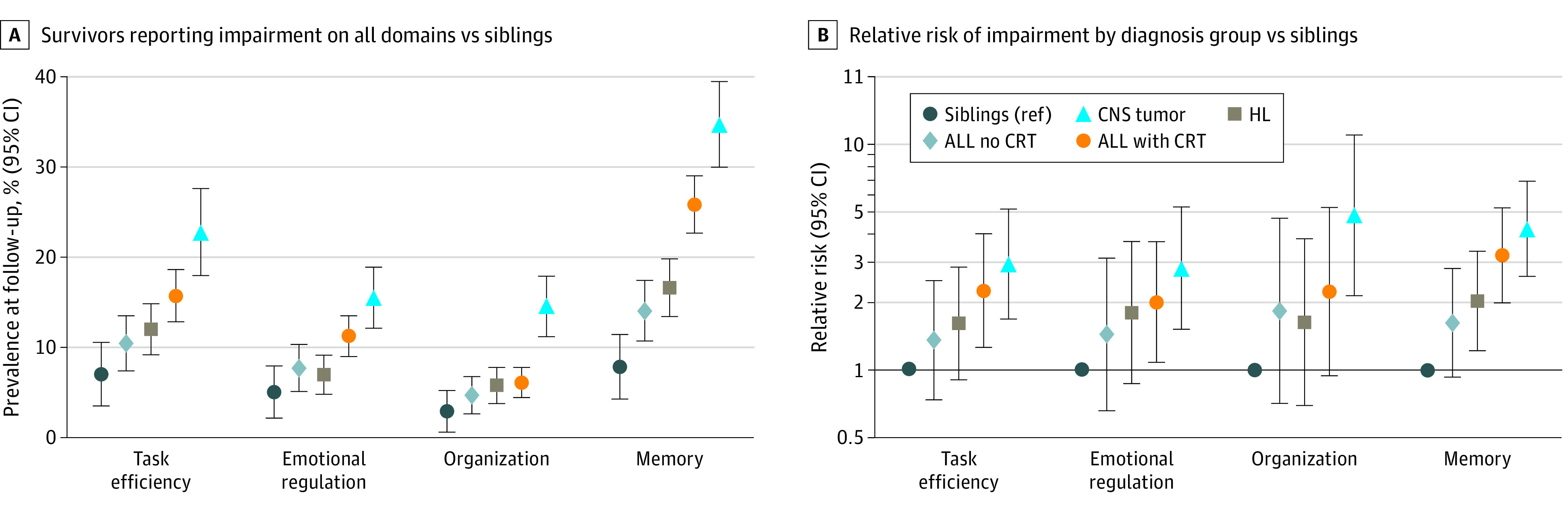
Prevalence and Relative Risk of Neurocognitive Impairment in Survivors Compared With Sibling Controls at Follow-up Cancer survivors who did not have impairment at baseline on a given cognitive domain demonstrate significantly greater risk of decline to cognitive impairment for that domain compared with sibling controls after an 11-year interval. Panel A shows that a higher percentage of survivors reported impairment on all domains at baseline compared with siblings. This increased rate of new-onset impairment can be seen in all measured cognitive domains. Panel B shows the relative risk of impairment by diagnosis group compared with siblings for all measured cognitive domains at follow-up among those not impaired on that measure at baseline, with 95% CIs adjusted for sex and age at baseline. Baseline was 23.4 years and follow-up was 35.0 since diagnosis. ALL indicates acute lymphoblastic leukemia; CNS, central nervous system; CRT, cranial radiation therapy; HL, Hodgkin lymphoma.

Among individuals reporting no memory impairment at baseline, survivors in every diagnosis and treatment group reported increased rates of memory impairment compared with siblings at follow-up: ALL chemotherapy only, 14.0% (95% CI, 10.7%-17.4%); ALL with CRT, 25.8% (95% CI, 22.6%-29.0%); CNS tumor, 34.7% (95% CI, 30.0%-39.5%); HL, 16.6% (95% CI, 13.4%-19.8%); and siblings, 7.8% (95% CI, 4.3%-11.4%) (Figure 2A and eTable 3 in Supplement 1). Compared with men, women were more likely to report new-onset memory impairment (ALL treated with CRT, RR, 1.48; 95% CI, 1.14-1.92; ALL chemotherapy only, RR, 2.18; 95% CI, 1.22-3.91; HL, RR, 2.45; 95% CI, 1.52-3.69) (Table 2). Survivors of ALL treated with CRT had elevated risk of new-onset task efficiency and emotional regulation impairments after adjusting for sex and age at baseline (Figure 2B). CNS tumor survivors reported elevated risk for new-onset impairments in all 4 domains.

**Table 2.** Multivariable Analysis of Sex and Treatment Factors Associated With New-Onset Neurocognitive Impairment at Follow-up Among Survivors With Unimpaired Neurocognitive Function at Baseline

Participant group	Neurocognitive outcomes, RR (95% CI)^a^			
	Memory	Emotional regulation	Task efficiency	Organization
ALL survivors treated with CRT (n = 861)				
No.	724	756	605	777
Female sex (vs male)	1.48 (1.14-1.92)^b^	1.02 (0.67-1.55)	1.17 (0.80-1.73)	1.02 (0.57-1.83)
Methotrexate administered IT and IV (reference, PO and/or IT)	1.48 (1.06-2.04)^b^	1.13 (0.66-1.91)	1.52 (0.95-2.44)	1.33 (0.62-2.85)
Methotrexate administered IT, IV, and IM (reference, PO and/or IT)	1.15 (0.75-1.77)	0.91 (0.44-1.85)	1.31 (0.72-2.39)	0.40 (0.12-1.36)
Methotrexate administered IT, IV, and PO (reference, PO and/or IT)	1.57 (1.01-2.46)^b^	0.73 (0.24-2.22)	1.04 (0.45-2.39)	1.17 (0.39-3.50)
Maximum CRT dose (≥20 Gy vs <20 Gy)^c^	1.16 (0.88-1.54)	1.35 (0.83-2.19)	1.44 (0.94-2.20)	1.61 (0.87-3.01)
Cytarabine (yes vs no)	NE	NE	NE	2.55 (1.22-5.35)^b^
ALL survivors treated with chemotherapy only (n = 455)				
No.	407	403	385	408
Female sex (vs male)	2.18 (1.22-3.91)^b^	2.48 (1.09-5.64)^b^	1.59 (0.83-3.05)	NE
Methotrexate administered IT and IV (reference, PO and/or IT)	0.92 (0.31-2.71)	0.71 (0.10-4.95)	0.72 (0.18-2.82)	NE
Methotrexate administered IT, IV, and IM (reference, PO and/or IT)	2.31 (1.10-4.83)^b^	1.89 (0.39-9.18)	0.71 (0.19-2.64)	NE
Methotrexate administered IT, IV, and PO (reference, PO and/or IT)	0.59 (0.28-1.27)	0.93 (0.41-2.13)	0.54 (0.23-1.28)	NE
Cytarabine (yes vs no)	0.47 (0.22-0.99)^b^	0.32 (0.13-0.76)^b^	0.62 (0.28-1.34)	NE
Alkylator CED <8000 mg/m^2^ (reference, none)	1.87 (0.84-4.18)	NE	NE	NE
Alkylator CED ≥8000 mg/m^2^ (reference, none)	2.80 (1.28-6.12)^b^	NE	NE	NE
Anthracycline ≥250 and alkylator ≥8000 mg/m^2^ (reference, anthracycline <250 and alkylators <8000 mg/m^2^)^d^	NE	NE	4.13 (1.26-13.6)^b^	NE
Anthracycline <250 or alkylator <8000 mg/m^2^ (reference, anthracycline <250 and alkylators <8000 mg/m^2^)^d^	NE	NE	3.12 (1.25-7.82)^b^	NE
CNS tumor survivors (n = 488)				
No.	386	439	290	421
Female sex (vs male)	1.13 (0.85-1.51)	0.91 (0.57-1.46)	1.34 (0.84-2.13)	1.47 (0.90-2.42)
Lomustine (yes vs no)	0.94 (0.60-1.48)	1.14 (0.58-2.27)	0.99 (0.51-1.93)	1.84 (0.92-3.68)
Craniospinal irradiation with focal boost (yes vs no RT)	1.97 (1.33-2.90)^b^	1.29 (0.69-2.40)	1.93 (1.04-3.58)^b^	1.21 (0.63-2.32)
Focal brain radiation (yes vs no RT)	1.60 (1.09-2.33)^b^	1.08 (0.60-1.94)	1.71 (0.98-2.98)	0.83 (0.44-1.58)
CRT (yes vs no RT)	1.17 (0.49-2.81)	2.30 (0.90-5.87)	1.29 (0.35-4.72)	NE
Ventricle to peritoneal shunt (yes vs no)	1.00 (0.74-1.37)	0.40 (0.22-0.75)^b^	0.88 (0.53-1.48)	1.03 (0.59-1.78)
HL survivors (n = 571)				
No.	518	520	510	523
Female sex (vs male)	2.45 (1.52-3.69)^b^	0.91 (0.47-1.78)	2.06 (1.18-3.59)^b^	1.68 (0.72-3.91)
Alkylator (yes vs no)	1.10 (0.70-1.73)	0.78 (0.35-1.74)	0.58 (0.34-0.99)^b^	1.50 (0.57-3.96)
Anthracycline (yes vs no)	0.75 (0.30-1.86)	2.61 (1.00-6.81)^b^	1.10 (0.45-2.67)	0.75 (0.16-3.56)
Bleomycin (yes vs no)	1.44 (0.67-3.13)	0.73 (0.30-1.76)	2.09 (0.93-4.69)	1.34 (0.27-6.67)
Lomustine (yes vs no)	0.96 (0.41-2.24)	0.48 (0.07-3.19)	1.08 (0.38-3.07)	2.99 (1.05-8.56)^b^
Chest radiation <35 Gy (reference, none)	0.75 (0.30-1.89)	0.51 (0.19-1.38)	1.13 (0.41-3.11)	2.06 (0.29-14.6)
Chest radiation ≥35 Gy (reference, none)	1.26 (0.52-3.02)	0.52 (0.19-1.43)	1.00 (0.35-2.83)	2.04 (0.27-15.3)

Abbreviations: CED, cyclophosphamide equivalent dose; CRT, cranial radiation therapy; IM, intramuscular; IT, intrathecal; IV, intravenous; NE, not estimable; PO, by mouth; RR, relative risk; RT, radiation therapy.

^a^ Models for each outcome are mutually adjusted for all variables shown in addition to age at baseline.

^b^ Denotes statistical significance at *P* < .05.

^c^ Maximum CRT was taken as the sum of the prescribed doses from all overlapping cranial fields.

^d^ Anthracycline and alkylator dose were collapsed in the task efficiency model for ALL with chemotherapy alone. There were too few events to have both drugs in the model.

### Treatment Exposures

CRT dose was not associated with new-onset impairment in survivors of ALL (Table 2). However, new-onset memory problems were associated with combined intrathecal, intravenous, and oral methotrexate (RR, 1.57; 95% CI, 1.01-2.46) compared with intrathecal and/or oral methotrexate only, after adjusting for sex and age at baseline. Intrathecal, intravenous, and intramuscular methotrexate (RR, 2.31; 95% CI, 1.10-4.83) and cumulative alkylator doses greater than or equal to 8000 mg/m^2^ (RR, 2.80; 95% CI, 1.28-6.12) were associated with new-onset memory problems in ALL survivors treated with chemotherapy only. Among CNS tumor survivors, new-onset memory impairment was associated with craniospinal irradiation (RR, 1.97; 95% CI, 1.33-2.90) and focal radiation (RR, 1.60; 95% CI, 1.09-2.33). The highest RRs for radiation dose and new-onset memory impairment were seen with radiation exposure greater than 50 Gy to the temporal lobe (RR, 2.05; 95% CI, 1.47-2.86) (eTable 4 in Supplement 1).

Anthracyclines were associated with new-onset impaired emotional regulation (RR, 2.61; 95% CI, 1.01-6.81) among HL survivors. Combined anthracycline and alkylator chemotherapy (anthracycline ≥250 mg/m^2^ plus alkylator ≥8 g/m^2^, RR, 4.13; 95% CI, 1.26-13.6; anthracycline <250 mg/m^2^ plus alkylator <8 g/m^2^, RR, 3.12; 95% CI, 1.25-7.82) was associated with impaired task efficiency in ALL survivors treated with chemotherapy only. New-onset organization impairment was associated with cytarabine among ALL survivors treated with CRT (RR, 2.55; 95% CI, 1.22-5.35) and with lomustine treatment among HL survivors (RR, 2.99; 95% CI, 1.05-8.56).

### Chronic Health Conditions

**Figure 3.**
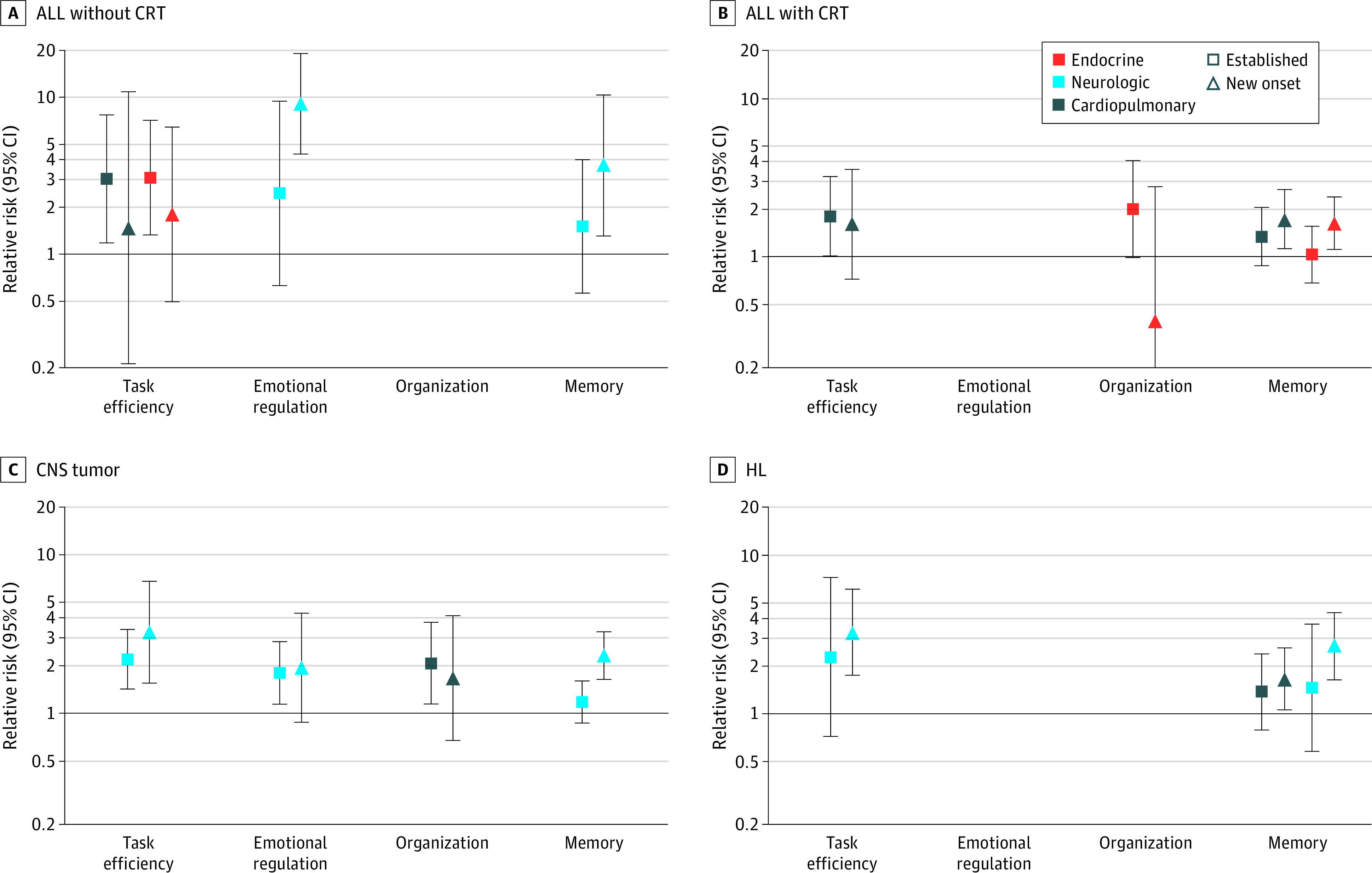
Relative Risk of New Onset Cognitive Impairment by Chronic Condition Onset Graphs show data for acute lymphoblastic leukemia (ALL) without cranial radiation therapy (CRT) (A), ALL with CRT (B), central nervous system (CNS) tumors (C), and Hodgkin lymphoma (HL) (D). Relative risk is based on models for conditions with significant results, adjusted for age and sex. Each model is shown for survivors who were unimpaired in the neurocognitive domain at baseline. Established indicates survivors with grade 3 or 4 condition at baseline; new onset indicates grade 3 or 4 condition developed between baseline and follow-up, with referent as no grade 3 or 4 condition by follow-up. Blank spaces indicate model for given domain had no significant chronic conditions.

Among survivors, the presence of chronic conditions at baseline was associated with new-onset impairment at follow-up. New-onset task efficiency impairment was associated with grade 3 and 4 cardiopulmonary conditions in ALL survivors (ALL CRT, RR, 1.83; 95% CI, 1.02-3.27; ALL chemotherapy RR, 3.04; 95% CI, 1.19-7.78), as well as with grade 3 and 4 endocrine conditions (ALL chemotherapy, RR, 3.10; 95% CI, 1.34-7.17) (Figure 3 and eTable 5 in Supplement 1). In CNS tumor survivors, grade 3 and 4 cardiopulmonary conditions were associated with new-onset organization impairment (RR, 2.07; 95% CI, 1.14-3.75), whereas neurologic conditions were associated with impaired emotional regulation (RR, 1.80; 95% CI, 1.14-2.83) and task efficiency (RR, 2.19; 95% CI, 1.42-3.38).

The development of grade 3 and 4 cardiopulmonary conditions after baseline was associated with new memory impairment in ALL survivors treated with CRT (RR, 1.74; 95% CI, 1.13-2.67) and HL survivors (RR, 1.66; 95% CI, 1.06-2.60), as well as task efficiency impairment in ALL survivors treated with chemotherapy only (RR, 3.04; 95% CI, 1.19-7.78). New onset of grade 3 and 4 neurologic conditions after baseline was associated with impaired memory (RR, 3.68; 95% CI, 1.30-10.4) and emotional regulation (RR, 9.14; 95% CI, 4.37-19.1) in ALL survivors, and impaired memory and task efficiency in both CNS tumor (memory RR, 2.32; 95% CI, 1.64-3.28; task efficiency RR, 3.25; 95% CI, 1.55-6.81) and HL (memory RR, 2.67; 95% CI, 1.64-4.35; task efficiency RR, 3.27; 95% CI, 1.75-6.14) survivors.

The association of anthracyclines and alkylators with impaired task efficiency among ALL survivors was partially mediated through cardiopulmonary conditions (estimate, 2.12; SE, 1.03; PM = 0.68; *P* = .04) (eTable 6 in Supplement 1). Among CNS tumor survivors, new-onset neurologic chronic conditions mediated the association of craniospinal irradiation with impaired memory (estimate, 2.02; SE, 1.01; PM = 0.78; *P* = .046) and task efficiency (estimate, 15.60; SE, 5.98; PM = 0.96; *P* = .008), as well as the association of focal radiation with impaired task efficiency (estimate, 17.04; SE, 6.74; PM = 0.98; *P* = .01).

### Health Behaviors

Being a smoker at baseline was associated with future risk for impaired memory (RR, 1.56; 95% CI, 1.11-2.18) and task efficiency (RR, 2.04; 95% CI, 1.30-3.19) in ALL survivors treated with CRT and with impaired emotional regulation in survivors of CNS tumor (RR, 2.34; 95% CI, 1.38-3.98) and HL (RR, 2.83; 95% CI, 1.26-6.35) (eTable 7 in Supplement 1). Among ALL survivors, lack of regular physical activity at baseline was associated with future impaired organization (RR, 1.82; 95% CI, 1.01-3.30) in those treated with CRT and with impaired emotional regulation (RR, 4.27; 95% CI, 1.95-9.36) in the chemotherapy-only group. For CNS tumor survivors, low baseline physical activity was associated with future impaired memory (RR, 1.43; 95% CI, 1.09-1.89), emotional regulation (RR, 1.72; 95% CI, 1.10-2.69), and organization (RR, 2.51; 95% CI, 1.53-4.10). In HL survivors, obesity at baseline was associated with future impaired memory (RR, 1.72; 95% CI, 1.11-2.65).

## Discussion

Approximately 40% of long-term survivors of childhood cancer demonstrate neurocognitive impairment 5 to 10 years after diagnosis.^6,7^ However, very-long-term risk for the 60% who do not experience early impairment has not been determined. To our knowledge, the current cohort study, which uses a large and well-characterized cohort of adult childhood cancer survivors, provides the first extensive evidence of substantial prevalence and risk factors for late-onset neurocognitive impairments. Risk factors included original treatment exposures, modifiable health behaviors, and chronic health conditions. Few chronic conditions mediated the impact of the original cancer treatment on neurocognitive outcomes, suggesting a lifelong risk associated with treatment exposure. This pattern, and the fact that memory impairment was most common, suggests that the original cancer therapies are associated with physiological changes that accelerate aging or the rate of neurocognitive decline.^28^

CNS-directed therapies administered early in life alter the typical course of brain development and are associated with reduced cortical thickness and altered network connectivity.^29,30^ Cancer-related therapies can cause biological changes that lead to cerebrovascular damage, stem cell depletion or mutation, and oxidative stress and inflammation.^31^ Ionizing radiation causes cellular senescence, epigenetic alterations, and DNA disrepair.^32^ Methotrexate causes epigenetic alteration and inhibits free radical reduction.^33,34^ It may be through these mechanisms that adult survivors of childhood cancer are at increased risk for reduced neurocognitive reserve, which would offer less resilience against accumulated life stressors and accelerate age-related decline.^35^

We found that severe or disabling and life-threatening cardiopulmonary and neurologic chronic health conditions carry significant independent risk for neurocognitive impairment. The importance of this association cannot be overstated given that cancer survivors 26 years from diagnosis carry a 15-fold excess risk of cardiovascular morbidities compared with similarly aged persons in the general population.^18^ Furthermore, despite strong recommendations for screening and early intervention for many of these late effects, only 41% of survivors report adherence to the recommended guidelines.^36^ Thus, we can anticipate that without improvements in early interventions, the number of survivors developing neurocognitive impairments will increase as the survivor population expands and ages. This will put additional financial and emotional pressure on families of cancer survivors, who may have limited resources available to care for an aging, cognitively impaired family member.

Alkylators, such as cyclophosphamide, can induce primary ovarian insufficiency and early menopause,^37^ which may increase the risk of cardiovascular and neurologic conditions such as atherosclerosis and stroke in women.^38^ Moreover, estrogen has a beneficial effect on the aging brain, and reduced estradiol production is associated with decreased working memory function in older adult women.^39^ Hypogonadism induced by alkylators may play some role in accelerated memory impairments seen in this study, as demonstrated by the high RR associated with female sex. No association was found with endocrine chronic conditions, but the approach for using self-reported information for severity grading in the CCSS cohort currently does not ascertain the impact of oral contraceptives and hormone replacement and, as such, may not accurately capture the risk for premature menopause previously described in this cohort.^40,41^

A unique strength of the current analysis was the ability to consider sociodemographic and health behaviors. The risk of neurocognitive impairment at follow-up was associated with obesity, smoking, decreased physical activity, and limited educational attainment at baseline. This finding implies that lifestyle choices made early in survivorship are associated with neurocognitive outcomes a decade later. This is similar to what is seen in populations without cancer, with those having ever (vs never) smoked in childhood carrying a greater risk for inferior neurocognitive outcomes.^42^ These modifiable risk factors often carry a greater RR than some treatment factors. Our findings are consistent with risks seen in populations without cancer, where smoking, reduced physical activity, and poor education were associated with increased risk for accelerated neurocognitive impairments,^43^ albeit at an older age compared with cancer survivors. This risk may be of greater consequence among cancer survivors who report more symptoms of risky health behaviors in association with poor chronic health conditions.^44^ Our findings support prioritizing future interventions that include strategies to increase physical activity and prevent smoking early in the life of survivors, as the presence of these conditions can have a profound risk for survivors later in life.

### Limitations

There are several limitations to be considered when interpreting the results of this study. First, the survivors in this study were treated using historic protocols that may not represent current therapies. However, as this is the cohort of survivors who are currently reaching the age when the risk for accelerated cognitive aging is the greatest, it is important to identify risk factors for this group so that screening and interventions can be implemented. Second, some concerns may be raised that the small sibling cohort may not adequately represent the population without cancer. Previous studies^45,46^ from CCSS have demonstrated that siblings are at no greater risk for neurocognitive problems or chronic health conditions than other populations without cancer. Third, the chronic condition data were self-reported. Bearing this in mind, some chronic conditions, such as estrogen deficiency after puberty, cerebrovascular disease, or cardiomyopathy, may be underappreciated until late in the history of the disease. This can result in underreporting, and the relative risk of endocrine, neurologic, or cardiopulmonary diseases may be higher than reported here. Fourth, the survivors who completed surveys at both baseline and follow-up were more likely to be female, older at diagnosis, college educated, employed, and independent compared with those who only completed medical surveys (eTable 8 in Supplement 1). As a result, we may underestimate the risks associated with neurocognitive impairment in all survivors. Additionally, the CCSS-NCQ is a validated and well-respected instrument; however, it is not all-inclusive and cannot capture all potential neurocognitive problems survivors may experience. Self-report measures capture meaningful, overt, neurocognitive changes, whereas objective measures may be able to capture smaller changes over shorter periods. Moreover, potential misclassification based on survivor’s self-report would not account for the reported decline since this is a repeated measures test using the same instrument. Fifth, some groups of survivors may underappreciate their neurocognitive impairments and, as such, fail to report significant impairments over time.

## Conclusions

The findings of this cohort study emphasize that childhood cancer survivors may be at perennial risk of cognitive decline throughout their lives. Practitioners should consider neurocognitive surveillance for all survivors despite the lack of neurocognitive impairments at time of completion of therapy. Serial evaluations of neurocognitive function in every survivor, including those who did not receive CNS-directed therapies, and adherence to survivorship late-effects screening recommendations will be critical to maintaining cognitive function in aging cancer survivors. Although there are currently no treatments that reverse cognitive declines, health care practitioners may be able to reduce a survivor’s risk for accelerated neurocognitive decline by targeting modifiable risk factors (eg, obesity, smoking, and low physical activity) for early intervention (eg, structured exercise programs, smoking prevention, or cessation strategies, in combination with trials of bupropion and varenicline for current smokers) in childhood to reduce chronic health morbidities.

## Notes

Supplement 1. **eFigure 1.** Details for Childhood Cancer Survivors Who Met Eligibility Requirements for This Study **eFigure 2.** Consort Diagram of Childhood Cancer Siblings Who Met Eligibility Requirements for This Study **eTable 1.** Treatment Information for Survivors by Diagnosis Group and NCQ Domain in Those Who Were Unimpaired at Baseline **eTable 2.** Incidence of Chronic Condition by Diagnosis Group **eTable 3.** Prevalence and Relative Risk of Neurocognitive Impairment at Follow-up in Survivors Compared to Siblings Among Those With Unimpaired Function in the Domain at Baseline **eTable 4.** Univariate Models for Radiation Exposure by Dose in CNS Tumor Survivor and Risk for New-Onset Neurocognitive Impairment at Follow-up (T2) Among Those With No Impairment at Baseline (T1) **eTable 5.** Multivariable Analysis of Timing of Chronic Condition Onset and Relative Risk for New-Onset Neurocognitive Impairment in Cognitive Function Domains Among Survivors With No Impairment in the Domain at Baseline **eTable 6.** Effects of Treatment and Evaluating Mediation by Chronic Health Conditions on Late Onset of Cognitive Impairment Among Childhood Cancer Survivors **eTable 7.** Multivariable Analysis of Health Outcomes and Health Behaviors Associated With New-Onset Neurocognitive Impairment of the Measures in the CCSS-NCQ From Baseline to Follow-up Among Survivors With Unimpaired Neurocognitive Function in the Domain at Baseline **eTable 8.** Comparison of Survivors With Completed Baseline NCQ, Unimpaired on at Least 1 Scale, and Still Alive as of the Follow-up Mailing to Those Who Competed Both Baseline and Follow-up NCQ Questionnaires, by Diagnosis

Supplement 2.
Data Sharing Statement
