# Supplementary material for: Late-onset Cognitive Impairment and Modifiable Risk Factors in Adult Childhood Cancer Survivors
Source: JAMA Netw Open. 2023 May 31;6(5):e2316077. doi: 10.1001/jamanetworkopen.2023.16077 (PMC10233416; doi:10.1001/jamanetworkopen.2023.16077)
Supplement: Supplement 1. — eFigure 1. Details for Childhood Cancer Survivors Who Met Eligibility Requirements for This Study eFigure 2. Consort Diagram of Childhood Cancer Siblings Who Met Eligibility Requirements for This Study eTable 1. Treatment Information for Survivors by Diagnosis Group and NCQ Domain in Those Who Were Unimpaired at Baseline eTable 2. Incidence of Chronic Condition by Diagnosis Group eTable 3. Prevalence and Relative Risk of Neurocognitive Impairment at Follow-up in Survivors Compared to Siblings Among Those With Unimpaired Function in the Domain at Baseline eTable 4. Univariate Models for Radiation Exposure by Dose in CNS Tumor Survivor and Risk for New-Onset Neurocognitive Impairment at Follow-up (T2) Among Those With No Impairment at Baseline (T1) eTable 5. Multivariable Analysis of Timing of Chronic Condition Onset and Relative Risk for New-Onset Neurocognitive Impairment in Cognitive Function Domains Among Survivors With No Impairment in the Domain at Baseline eTable 6. Effects of Treatment and Evaluating Mediation by Chronic Health Conditions on Late Onset of Cognitive Impairment Among Childhood Cancer Survivors eTable 7. Multivariable Analysis of Health Outcomes and Health Behaviors Associated With New-Onset Neurocognitive Impairment of the Measures in the CCSS-NCQ From Baseline to Follow-up Among Survivors With Unimpaired Neurocognitive Function in the Domain at Baseline eTable 8. Comparison of Survivors With Completed Baseline NCQ, Unimpaired on at Least 1 Scale, and Still Alive as of the Follow-up Mailing to Those Who Competed Both Baseline and Follow-up NCQ Questionnaires, by Diagnosis [file jamanetwopen-e2316077-s001.pdf]

Supplemental Online Content

Phillips NS, Stratton KL, Williams AM, et al. Late-onset cognitive impairment and modifiable risk factors in adult childhood cancer survivors. *JAMA Netw Open*. 2023;6(5):e2316077. doi:10.1001/jamanetworkopen.2023.16077

eFigure 1. Details for Childhood Cancer Survivors Who Met Eligibility Requirements for This Study

eFigure 2. Consort Diagram of Childhood Cancer Siblings Who Met Eligibility Requirements for This Study

eTable 1. Treatment Information for Survivors by Diagnosis Group and NCQ Domain in Those Who Were Unimpaired at Baseline

eTable 2. Incidence of Chronic Condition by Diagnosis Group

eTable 3. Prevalence and Relative Risk of Neurocognitive Impairment at Follow-up in Survivors Compared to Siblings Among Those With Unimpaired Function in the Domain at Baseline

eTable 4. Univariate Models for Radiation Exposure by Dose in CNS Tumor Survivor and Risk for New-Onset Neurocognitive Impairment at Follow-up (T2) Among Those With No Impairment at Baseline (T1)

eTable 5. Multivariable Analysis of Timing of Chronic Condition Onset and Relative Risk for New-Onset Neurocognitive Impairment in Cognitive Function Domains Among Survivors With No Impairment in the Domain at Baseline

eTable 6. Effects of Treatment and Evaluating Mediation by Chronic Health Conditions on Late Onset of Cognitive Impairment Among Childhood Cancer Survivors

eTable 7. Multivariable Analysis of Health Outcomes and Health Behaviors Associated With New-Onset Neurocognitive Impairment of the Measures in the CCSS-NCQ From Baseline to Follow-up Among Survivors With Unimpaired Neurocognitive Function in the Domain at Baseline

eTable 8. Comparison of Survivors With Completed Baseline NCQ, Unimpaired on at Least 1 Scale, and Still Alive as of the Follow-up Mailing to Those Who Competed Both Baseline and Follow-up NCQ Questionnaires, by Diagnosis

This supplemental material has been provided by the authors to give readers additional information about their work.

© 2023 Phillips NS et al. *JAMA Network Open.*

eFigure 1. Details for Childhood Cancer Survivors Who Met Eligibility Requirements for This Study

3474 unimpaired on at least one neurocognitive scale at baseline and alive at follow-up mailing

745 CNS, **780** Hodgkin, **660** ALL no CRT, **1289** ALL w/CRT

→ -**158** refused, **66** lost to follow up, **73** other reasons

↓

3177 mailed a survey at follow-up			
664 CNS	725 Hodgkin	617 ALL no CRT	1171 ALL w/CRT
# unimpaired (T1):	# unimpaired (T1):	# unimpaired (T1):	# unimpaired (T1):
M 527	M 655	M 555	M 988
E 594	E 658	E 545	E 1028
T 384	T 637	T 511	T 825
O 571	O 655	O 547	O 1051

↓

**2375** completed at least one neurocognitive domain at follow-up			
488 CNS	571 Hodgkin	455 ALL no CRT	861 ALL w/CRT
# unimpaired (T1):	# unimpaired (T1):	# unimpaired (T1):	# unimpaired (T1):
M 386 (73%)	M 518 (79%)	M 407 (73%)	M 724 (73%)
E 439 (74%)	E 520 (79%)	E 403 (74%)	E 756 (74%)
T 290 (76%)	T 510 (80%)	T 385 (75%)	T 605 (73%)
O 421 (74%)	O 523 (80%)	O 408 (75%)	O 777 (74%)

Unimpaired (T1) indicates number of survivors unimpaired at baseline for each scale, with

M=memory, E=emotional regulation, T=task efficiency and O=organization.

© 2023 Phillips NS et al. *JAMA Network Open.*

eFigure 2. Consort Diagram of Childhood Cancer Siblings Who Met Eligibility Requirements for This Study

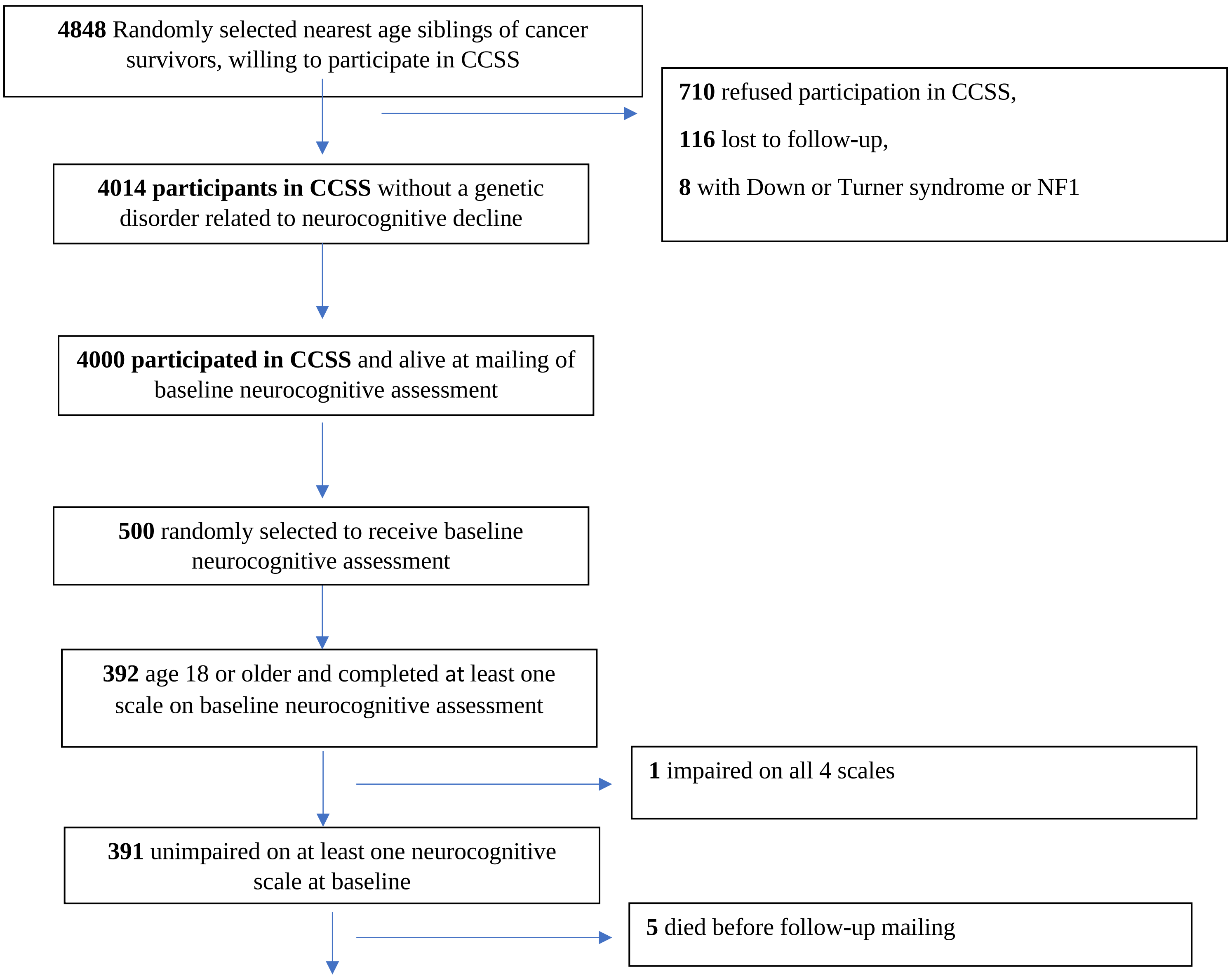

© 2023 Phillips NS et al. *JAMA Network Open.*

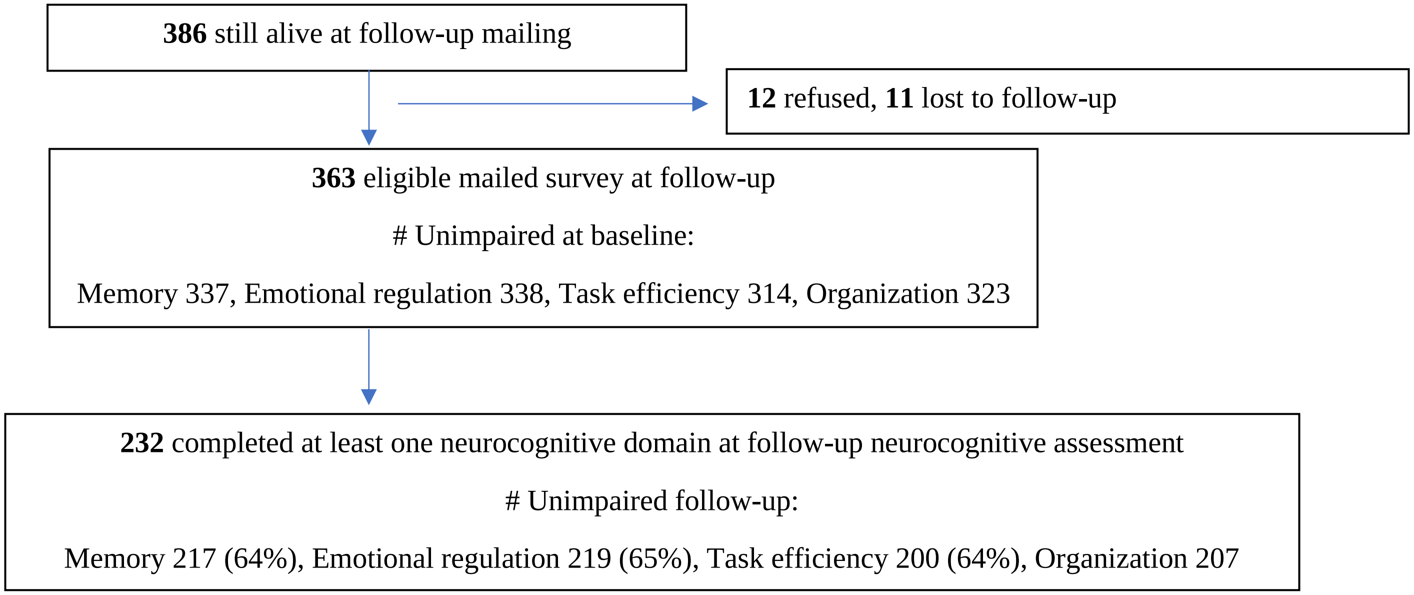

© 2023 Phillips NS et al. *JAMA Network Open.*

eTable 1. Treatment Information for Survivors by Diagnosis Group and NCQ Domain in Those Who Were Unimpaired at Baseline

			NCQ			
			Memory	Emotion	Task efficiency	Organization
Diagnosis group			N (%)	N (%)	N (%)	N (%)
ALL no CRT	Total		407 (100)	403 (100)	385 (100)	408 (100)
	Medical record abstraction	Yes	406 (99.8)	402 (99.8)	384 (99.7)	407 (99.8)
	Any anthracycline	Yes	129 (31.9)	132 (32.9)	127 (33.1)	132 (32.5)
	Any alkylator	Yes	202 (50.0)	204 (51.0)	193 (50.3)	202 (49.8)
	IV methotrexate	Yes	202 (50.1)	199 (49.9)	195 (50.9)	210 (51.7)
	IT methotrexate	Yes	369 (91.6)	366 (91.5)	352 (91.9)	373 (92.1)
	IM methotrexate	Yes	38 (9.4)	32 (8.0)	36 (9.4)	43 (10.6)
	Ara-C IV	Yes	114 (28.1)	114 (28.4)	116 (30.2)	121 (29.7)
	Ara-C SubQ	Yes	12 (3.0)	12 (3.0)	10 (2.6)	12 (2.9)
	Any Ara-C	Yes	214 (52.8)	214 (53.4)	207 (53.9)	220 (54.1)
	Dexamethasone	Yes	36 (9.0)	36 (9.2)	37 (9.9)	40 (10.1)
	Prednisone	Yes	356 (96.0)	352 (96.4)	338 (96.6)	360 (96.8)
	Vincristine	Yes	357 (87.9)	352 (87.6)	338 (88.0)	361 (88.7)
	BCNU	Yes	36 (8.9)	37 (9.2)	36 (9.4)	37 (9.1)

© 2023 Phillips NS et al. *JAMA Network Open.*

			NCQ			
			**Memory**	**Emotion**	**Task efficiency**	**Organization**
**Diagnosis group**			**N (%)**	**N (%)**	**N (%)**	**N (%)**
	CCNU	Yes	0 (0)	1 (0.2)	1 (0.3)	1 (0.2)
	IV cyclophosphamide	Yes	175 (43.2)	176 (43.9)	167 (43.5)	175 (43.1)
	Hexamethylmelamine	Yes	1 (0.2)	1 (0.3)	1 (0.3)	1 (0.2)
	Methotrexate route	IT+IV	32 (7.9)	29 (7.3)	29 (7.6)	30 (7.4)
		IT+IV+IM	30 (7.4)	26 (6.5)	29 (7.6)	35 (8.6)
		IT+IV+PO	116 (28.8)	120 (30.0)	115 (30.0)	121 (29.9)
		IT+PO or PO only	173 (42.9)	175 (43.8)	163 (42.6)	169 (41.7)
		Other	52 (12.9)	50 (12.5)	47 (12.3)	50 (12.3)
	Any radiation	Yes	15 (3.7)	19 (4.7)	16 (4.2)	17 (4.2)
	Chest RT dose	Yes	2 (0.5)	3 (0.7)	3 (0.8)	3 (0.7)
	Direct Neck RT	Yes	2 (0.5)	3 (0.7)	3 (0.8)	3 (0.7)
	Direct abdomen RT	Yes	2 (0.5)	2 (0.5)	2 (0.5)	2 (0.5)
	Cumulative anthracycline dose (doxo-equivalent)	None	276 (69.9)	269 (68.8)	257 (68.4)	274 (69.2)
		<100 mg	41 (10.4)	40 (10.2)	41 (10.9)	42 (10.6)

© 2023 Phillips NS et al. *JAMA Network Open.*

			NCQ			
			Memory	Emotion	Task efficiency	Organization
**Diagnosis group**			**N (%)**	**N (%)**	**N (%)**	**N (%)**
		100-249 mg	54 (13.7)	56 (14.3)	53 (14.1)	55 (13.9)
		250+ mg	24 (6.1)	26 (6.6)	25 (6.6)	25 (6.3)
	Cumulative alkylator dose (cyclo-equivalent)	None	202 (51.8)	196 (51.0)	191 (51.5)	204 (52.2)
		<4000 mg/m2	107 (27.4)	107 (27.9)	100 (27.0)	105 (26.9)
		4000-7999 mg/m2	23 (5.9)	25 (6.5)	21 (5.7)	24 (6.1)
		8000+ mg/m2	58 (14.9)	56 (14.6)	59 (15.9)	58 (14.8)
	Cumulative platinum dose (cisplatin-equivalent)	None	406 (100)	402 (100)	384 (100)	407 (100)
	IV methotrexate dose	None	201 (50.9)	200 (51.3)	188 (50.0)	196 (49.4)
		<4300 mg/m2	84 (21.3)	83 (21.3)	84 (22.3)	81 (20.4)
		4300+ mg/m2	110 (27.8)	107 (27.4)	104 (27.7)	120 (30.2)
	Chest RT dose	None	392 (96.3)	384 (95.3)	369 (95.8)	391 (95.8)
		<10 Gy	13 (3.2)	16 (4.0)	13 (3.4)	14 (3.4)
		10-19 Gy	1 (0.2)	1 (0.2)	1 (0.3)	1 (0.2)

© 2023 Phillips NS et al. *JAMA Network Open.*

			NCQ			
			Memory	Emotion	Task efficiency	Organization
**Diagnosis group**			**N (%)**	**N (%)**	**N (%)**	**N (%)**
		20-29 Gy	1 (0.2)	2 (0.5)	2 (0.5)	2 (0.5)
	Pelvic RT dose	None	392 (96.3)	384 (95.3)	369 (95.8)	391 (95.8)
		<10 Gy	15 (3.7)	19 (4.7)	16 (4.2)	17 (4.2)
	Abdomen RT dose	None	392 (96.3)	384 (95.3)	369 (95.8)	391 (95.8)
		<10 Gy	13 (3.2)	17 (4.2)	14 (3.6)	15 (3.7)
		10-19 Gy	1 (0.2)	1 (0.2)	1 (0.3)	1 (0.2)
		30-39 Gy	1 (0.2)	1 (0.2)	1 (0.3)	1 (0.2)
	Neck RT dose	None	392 (96.3)	384 (95.3)	369 (95.8)	391 (95.8)
		<10 Gy	13 (3.2)	17 (4.2)	14 (3.6)	15 (3.7)
		20-29 Gy	2 (0.5)	2 (0.5)	2 (0.5)	2 (0.5)
	TBI dose	None	407 (100)	403 (100)	385 (100)	408 (100)
	Max brain RT dose	None	392 (96.3)	384 (95.3)	369 (95.8)	391 (95.8)
		<10 Gy	15 (3.7)	19 (4.7)	16 (4.2)	17 (4.2)
	Infratentorial RT dose	None	392 (96.3)	384 (95.3)	369 (95.8)	391 (95.8)
		<10 Gy	15 (3.7)	19 (4.7)	16 (4.2)	17 (4.2)

© 2023 Phillips NS et al. *JAMA Network Open.*

			NCQ			
			Memory	Emotion	Task efficiency	Organization
**Diagnosis group**			**N (%)**	**N (%)**	**N (%)**	**N (%)**
	Temporal lobe RT dose	None	392 (96.3)	384 (95.3)	369 (95.8)	391 (95.8)
		<10 Gy	15 (3.7)	19 (4.7)	16 (4.2)	17 (4.2)
	Frontal lobe RT dose	None	392 (96.3)	384 (95.3)	369 (95.8)	391 (95.8)
		<10 Gy	15 (3.7)	19 (4.7)	16 (4.2)	17 (4.2)
	Parietal/Occipital RT dose	None	392 (96.3)	384 (95.3)	369 (95.8)	391 (95.8)
		<10 Gy	15 (3.7)	19 (4.7)	16 (4.2)	17 (4.2)
	CNS surgery including shunt	Yes	10 (2.5)	10 (2.5)	10 (2.6)	10 (2.5)
ALL w/CRT	Total		724 (100)	756 (100)	605 (100)	777 (100)
	Medical record abstraction	Yes	713 (98.5)	745 (98.5)	596 (98.5)	765 (98.5)
	Any anthracycline	Yes	362 (50.9)	374 (50.3)	300 (50.4)	381 (49.9)
	Any alkylator	Yes	345 (48.7)	371 (50.1)	283 (47.6)	375 (49.3)
	IV methotrexate	Yes	238 (33.5)	245 (33.0)	188 (31.6)	254 (33.3)
	IT methotrexate	Yes	650 (91.7)	677 (91.4)	544 (91.6)	694 (91.2)
	IM methotrexate	Yes	82 (11.5)	83 (11.2)	66 (11.1)	88 (11.5)
	Ara-C IV	Yes	283 (39.9)	292 (39.4)	226 (38.1)	300 (39.4)

© 2023 Phillips NS et al. *JAMA Network Open.*

			NCQ			
			Memory	Emotion	Task efficiency	Organization
**Diagnosis group**			**N (%)**	**N (%)**	**N (%)**	**N (%)**
	Ara-C SubQ	Yes	66 (9.3)	70 (9.4)	53 (8.9)	63 (8.2)
	Any Ara-C	Yes	342 (48.2)	356 (48.0)	276 (46.5)	366 (48.0)
	Dexamethasone	Yes	71 (10.2)	79 (10.8)	63 (10.8)	78 (10.4)
	Prednisone	Yes	566 (97.6)	590 (97.0)	471 (97.3)	609 (97.3)
	IV Bleomycin	Yes	1 (0.1)	1 (0.1)	1 (0.2)	1 (0.1)
	Vincristine	Yes	569 (79.8)	592 (79.5)	474 (79.5)	612 (80.0)
	BCNU	Yes	15 (2.1)	15 (2.0)	14 (2.3)	16 (2.1)
	CCNU	Yes	1 (0.1)	1 (0.1)	1 (0.2)	1 (0.1)
	IV cyclophosphamide	Yes	285 (40.2)	299 (40.4)	224 (37.7)	303 (39.8)
	Melphalan	Yes	0 (0)	1 (0.1)	0 (0)	1 (0.1)
	Nitrogen mustard	Yes	1 (0.1)	1 (0.1)	1 (0.2)	1 (0.1)
	Methotrexate route	IT+IV	114 (16.1)	123 (16.6)	89 (15.0)	122 (16.0)
		IT+IV+IM	76 (10.7)	77 (10.4)	60 (10.1)	82 (10.8)
		IT+IV+PO	42 (5.9)	38 (5.1)	34 (5.7)	43 (5.7)
		IT+PO or PO only	378 (53.3)	392 (52.9)	321 (54.0)	410 (53.9)

© 2023 Phillips NS et al. *JAMA Network Open.*

			NCQ			
			**Memory**	**Emotion**	**Task efficiency**	**Organization**
**Diagnosis group**			**N (%)**	**N (%)**	**N (%)**	**N (%)**
		Other	99 (14.0)	111 (15.0)	90 (15.2)	104 (13.7)
	Any radiation	Yes	724 (100)	756 (100)	605 (100)	777 (100)
	Direct Brain RT	Yes	711 (98.2)	744 (98.4)	594 (98.2)	766 (98.6)
	Chest RT dose	Yes	120 (16.6)	125 (16.6)	101 (16.7)	129 (16.6)
	Direct Neck RT	Yes	110 (15.2)	117 (15.5)	91 (15.1)	120 (15.5)
	Direct abdomen RT	Yes	109 (15.1)	117 (15.5)	89 (14.7)	118 (15.2)
	Direct Pelvic RT	Yes	110 (15.2)	119 (15.8)	90 (14.9)	121 (15.6)
	TBI	Yes	24 (3.3)	23 (3.1)	20 (3.3)	22 (2.8)
	Cumulative anthracycline dose (dox-equivalent)	None	349 (51.3)	369 (51.8)	295 (51.5)	382 (52.0)
		<100 mg	101 (14.9)	105 (14.7)	85 (14.8)	105 (14.3)
		100-249 mg	135 (19.9)	142 (19.9)	112 (19.5)	149 (20.3)
		250+ mg	95 (14.0)	97 (13.6)	81 (14.1)	98 (13.4)
	Cumulative alkylator dose (cyclo-equivalent)	None	365 (54.6)	371 (53.0)	312 (55.4)	387 (53.7)
		<4000 mg/m2	118 (17.6)	128 (18.3)	96 (17.1)	121 (16.8)

© 2023 Phillips NS et al. *JAMA Network Open.*

			NCQ			
			**Memory**	**Emotion**	**Task efficiency**	**Organization**
**Diagnosis group**			**N (%)**	**N (%)**	**N (%)**	**N (%)**
		4000-7999 mg/m2	70 (10.5)	75 (10.7)	60 (10.7)	82 (11.4)
		8000+ mg/m2	116 (17.3)	126 (18.0)	95 (16.9)	131 (18.2)
	Cumulative platinum dose (cisplatin-equivalent)	None	713 (100)	745 (100)	596 (100)	765 (100)
	IV methotrexate dose	None	473 (68.0)	498 (68.0)	406 (69.6)	509 (68.1)
		<4300 mg/m2	195 (28.0)	202 (27.6)	156 (26.8)	207 (27.7)
		4300+ mg/m2	28 (4.0)	32 (4.4)	21 (3.6)	31 (4.1)
	Chest RT dose	<10 Gy	592 (81.9)	619 (82.2)	492 (81.6)	639 (82.6)
		10-19 Gy	66 (9.1)	65 (8.6)	54 (9.0)	66 (8.5)
		20-29 Gy	60 (8.3)	65 (8.6)	54 (9.0)	64 (8.3)
		30-39 Gy	5 (0.7)	4 (0.5)	3 (0.5)	5 (0.6)
	Pelvic RT dose	<10 Gy	602 (83.3)	625 (83.0)	502 (83.3)	647 (83.6)
		10-19 Gy	62 (8.6)	65 (8.6)	50 (8.3)	64 (8.3)
		20-29 Gy	53 (7.3)	58 (7.7)	48 (8.0)	57 (7.4)
		30-39 Gy	6 (0.8)	5 (0.7)	3 (0.5)	6 (0.8)

© 2023 Phillips NS et al. *JAMA Network Open.*

			NCQ			
			**Memory**	**Emotion**	**Task efficiency**	**Organization**
**Diagnosis group**			**N (%)**	**N (%)**	**N (%)**	**N (%)**
	Abdomen RT dose	<10 Gy	602 (83.3)	626 (83.1)	502 (83.3)	649 (83.9)
		10-19 Gy	61 (8.4)	63 (8.4)	49 (8.1)	61 (7.9)
		20-29 Gy	55 (7.6)	60 (8.0)	50 (8.3)	59 (7.6)
		30-39 Gy	5 (0.7)	4 (0.5)	2 (0.3)	5 (0.6)
	Neck RT dose	<10 Gy	599 (82.8)	624 (82.9)	499 (82.8)	645 (83.3)
		10-19 Gy	55 (7.6)	56 (7.4)	43 (7.1)	55 (7.1)
		20-29 Gy	64 (8.9)	69 (9.2)	58 (9.6)	69 (8.9)
		30-39 Gy	5 (0.7)	4 (0.5)	3 (0.5)	5 (0.6)
	TBI dose	None	699 (96.7)	730 (96.9)	583 (96.7)	752 (97.2)
		<10 Gy	3 (0.4)	3 (0.4)	2 (0.3)	4 (0.5)
		10-19 Gy	21 (2.9)	20 (2.7)	18 (3.0)	18 (2.3)
	Max Brain RT dose	<10 Gy	1 (0.1)	1 (0.1)	1 (0.2)	1 (0.1)
		10-19 Gy	327 (45.4)	338 (45.1)	275 (45.8)	348 (45.1)
		20-29 Gy	361 (50.1)	382 (50.9)	303 (50.5)	394 (51.1)
		30-39 Gy	20 (2.8)	19 (2.5)	16 (2.7)	19 (2.5)

© 2023 Phillips NS et al. *JAMA Network Open.*

			NCQ			
			Memory	Emotion	Task efficiency	Organization
Diagnosis group			N (%)	N (%)	N (%)	N (%)
		40-49 Gy	10 (1.4)	10 (1.3)	5 (0.8)	8 (1.0)
		50+ Gy	1 (0.1)	0 (0)	0 (0)	1 (0.1)
	Infratentorial RT dose	<10 Gy	1 (0.1)	1 (0.1)	1 (0.2)	1 (0.1)
		10-19 Gy	327 (45.4)	338 (45.1)	275 (45.8)	348 (45.1)
		20-29 Gy	361 (50.1)	382 (50.9)	303 (50.5)	394 (51.1)
		30-39 Gy	20 (2.8)	19 (2.5)	16 (2.7)	19 (2.5)
		40-49 Gy	10 (1.4)	10 (1.3)	5 (0.8)	8 (1.0)
		50+ Gy	1 (0.1)	0 (0)	0 (0)	1 (0.1)
	Temporal lobe RT dose	<10 Gy	1 (0.1)	1 (0.1)	1 (0.2)	1 (0.1)
		10-19 Gy	327 (45.4)	338 (45.1)	275 (45.8)	348 (45.1)
		20-29 Gy	361 (50.1)	382 (50.9)	303 (50.5)	394 (51.1)
		30-39 Gy	20 (2.8)	19 (2.5)	16 (2.7)	19 (2.5)
		40-49 Gy	10 (1.4)	10 (1.3)	5 (0.8)	8 (1.0)
		50+ Gy	1 (0.1)	0 (0)	0 (0)	1 (0.1)
	Frontal lobe RT dose	<10 Gy	1 (0.1)	1 (0.1)	1 (0.2)	1 (0.1)

© 2023 Phillips NS et al. *JAMA Network Open.*

			NCQ			
			Memory	Emotion	Task efficiency	Organization
Diagnosis group			N (%)	N (%)	N (%)	N (%)
		10-19 Gy	328 (45.6)	338 (45.1)	275 (45.8)	349 (45.3)
		20-29 Gy	360 (50.0)	382 (50.9)	303 (50.5)	393 (51.0)
		30-39 Gy	21 (2.9)	19 (2.5)	16 (2.7)	20 (2.6)
		40-49 Gy	10 (1.4)	10 (1.3)	5 (0.8)	8 (1.0)
	Parietal/Occipital lobe RT dose	<10 Gy	1 (0.1)	1 (0.1)	1 (0.2)	1 (0.1)
		10-19 Gy	328 (45.6)	338 (45.1)	275 (45.8)	349 (45.3)
		20-29 Gy	360 (50.0)	382 (50.9)	303 (50.5)	393 (51.0)
		30-39 Gy	21 (2.9)	19 (2.5)	16 (2.7)	20 (2.6)
		40-49 Gy	10 (1.4)	10 (1.3)	5 (0.8)	8 (1.0)
	CNS surgery including shunt	Yes	12 (1.7)	10 (1.3)	7 (1.2)	12 (1.6)
CNS	Total		386 (100)	439 (100)	290 (100)	421 (100)
	Diagnosis	Astrocytoma	238 (61.7)	270 (61.5)	191 (65.9)	256 (60.8)
		Medulloblastoma, PNET	84 (21.8)	94 (21.4)	52 (17.9)	93 (22.1)
		Other CNS tumors	64 (16.6)	75 (17.1)	47 (16.2)	72 (17.1)

© 2023 Phillips NS et al. *JAMA Network Open.*

			NCQ			
			Memory	Emotion	Task efficiency	Organization
Diagnosis group			N (%)	N (%)	N (%)	N (%)
	Medical record abstraction	Yes	369 (95.6)	423 (96.4)	281 (96.9)	406 (96.4)
	Any platinum drug	Yes	21 (5.7)	25 (5.9)	12 (4.3)	23 (5.7)
	Any anthracycline	Yes	2 (0.5)	2 (0.5)	2 (0.7)	2 (0.5)
	Any alkylator	Yes	55 (14.9)	64 (15.1)	36 (12.8)	65 (16.0)
	IV methotrexate	Yes	3 (0.8)	2 (0.5)	1 (0.4)	3 (0.7)
	IT methotrexate	Yes	5 (1.4)	4 (0.9)	1 (0.4)	4 (1.0)
	Ara-C IV	Yes	11 (3.0)	15 (3.5)	7 (2.5)	12 (3.0)
	Any Ara-C	Yes	12 (3.3)	15 (3.5)	8 (2.8)	13 (3.2)
	Dexamethasone	Yes	23 (6.3)	22 (5.2)	15 (5.4)	21 (5.2)
	Prednisone	Yes	32 (8.7)	36 (8.5)	20 (7.1)	35 (8.6)
	IV Bleomycin	Yes	1 (0.3)	1 (0.2)	1 (0.4)	1 (0.2)
	Vincristine	Yes	57 (15.4)	65 (15.4)	35 (12.5)	62 (15.3)
	BCNU	Yes	2 (0.5)	3 (0.7)		5 (1.2)
	CCNU	Yes	38 (10.3)	45 (10.6)	29 (10.3)	45 (11.1)
	IV cyclophosphamide	Yes	12 (3.3)	15 (3.5)	4 (1.4)	12 (3.0)

© 2023 Phillips NS et al. *JAMA Network Open.*

			NCQ			
			Memory	Emotion	Task efficiency	Organization
**Diagnosis group**			**N (%)**	**N (%)**	**N (%)**	**N (%)**
	Nitrogen mustard	Yes	7 (1.9)	7 (1.7)	4 (1.4)	8 (2.0)
	Procarbazine	Yes	30 (8.1)	34 (8.0)	20 (7.1)	31 (7.6)
	DTIC	Yes	4 (1.1)	6 (1.4)	4 (1.4)	5 (1.2)
	Methotrexate route	IT+IV	1 (0.3)			1 (0.2)
		IT+PO or PO only	362 (98.1)	417 (98.6)	279 (99.3)	400 (98.5)
		Other	6 (1.6)	6 (1.4)	2 (0.7)	5 (1.2)
	Any radiation	Yes	226 (61.2)	271 (63.9)	164 (58.6)	259 (63.5)
	Direct Brain RT	Yes	204 (57.1)	247 (60.2)	148 (54.4)	237 (59.7)
	Chest RT dose	Yes	92 (25.8)	109 (26.6)	62 (22.8)	108 (27.2)
	Direct Neck RT	Yes	96 (26.9)	113 (27.6)	65 (23.9)	113 (28.5)
	Direct abdomen RT	Yes	94 (26.3)	112 (27.3)	64 (23.5)	110 (27.7)
	Direct Pelvic RT	Yes	91 (25.5)	107 (26.1)	62 (22.8)	106 (26.7)
	Cumulative anthracycline dose (doo-equivalent)	None	367 (99.7)	421 (99.8)	279 (99.6)	404 (99.8)
		250+ mg	1 (0.3)	1 (0.2)	1 (0.4)	1 (0.2)

© 2023 Phillips NS et al. *JAMA Network Open.*

			NCQ			
			**Memory**	**Emotion**	**Task efficiency**	**Organization**
**Diagnosis group**			**N (%)**	**N (%)**	**N (%)**	**N (%)**
	Cumulative alkylator dose(cyclo-equivalent)	None	314 (87.0)	359 (86.9)	245 (89.4)	341 (86.5)
		<4000 mg/m2	5 (1.4)	5 (1.2)	5 (1.8)	4 (1.0)
		4000-7999 mg/m2	6 (1.7)	6 (1.5)	3 (1.1)	6 (1.5)
		8000+ mg/m2	36 (10.0)	43 (10.4)	21 (7.7)	43 (10.9)
	Cumulative platinum dose (cisplatin-equivalent)	None	348 (94.6)	398 (94.3)	269 (96.1)	383 (94.6)
		<400 mg	9 (2.4)	11 (2.6)	6 (2.1)	12 (3.0)
		400+ mg	11 (3.0)	13 (3.1)	5 (1.8)	10 (2.5)
	IV methotrexate dose	None	366 (99.2)	421 (99.5)	280 (99.6)	403 (99.3)
		<4300 mg/m2	2 (0.5)	1 (0.2)	0 (0)	2 (0.5)
		4300+ mg/m2	1 (0.3)	1 (0.2)	1 (0.4)	1 (0.2)
	Chest RT dose	None	143 (40.3)	153 (37.4)	116 (42.8)	149 (37.6)
		<10 Gy	125 (35.2)	151 (36.9)	95 (35.1)	143 (36.1)
		10-19 Gy	2 (0.6)	2 (0.5)	0 (0)	2 (0.5)

© 2023 Phillips NS et al. *JAMA Network Open.*

			NCQ			
			**Memory**	**Emotion**	**Task efficiency**	**Organization**
**Diagnosis group**			**N (%)**	**N (%)**	**N (%)**	**N (%)**
		20-29 Gy	20 (5.6)	24 (5.9)	16 (5.9)	23 (5.8)
		30-39 Gy	51 (14.4)	63 (15.4)	36 (13.3)	62 (15.7)
		40-49 Gy	14 (3.9)	15 (3.7)	8 (3.0)	16 (4.0)
		50+ Gy	0 (0)	1 (0.2)	0 (0)	1 (0.3)
	Pelvic RT dose	None	143 (40.3)	153 (37.4)	116 (42.8)	149 (37.6)
		<10 Gy	126 (35.5)	153 (37.4)	95 (35.1)	145 (36.6)
		10-19 Gy	2 (0.6)	2 (0.5)	0 (0)	2 (0.5)
		20-29 Gy	21 (5.9)	25 (6.1)	17 (6.3)	24 (6.1)
		30-39 Gy	50 (14.1)	62 (15.2)	34 (12.5)	60 (15.2)
		40-49 Gy	10 (2.8)	12 (2.9)	6 (2.2)	13 (3.3)
		50+ Gy	3 (0.8)	2 (0.5)	3 (1.1)	3 (0.8)
	Abdomen RT dose	None	143 (40.3)	153 (37.4)	116 (42.8)	149 (37.6)
		<10 Gy	123 (34.6)	148 (36.2)	93 (34.3)	141 (35.6)
		10-19 Gy	2 (0.6)	2 (0.5)	0 (0)	2 (0.5)
		20-29 Gy	21 (5.9)	25 (6.1)	17 (6.3)	24 (6.1)

© 2023 Phillips NS et al. *JAMA Network Open.*

			NCQ			
			**Memory**	**Emotion**	**Task efficiency**	**Organization**
**Diagnosis group**			**N (%)**	**N (%)**	**N (%)**	**N (%)**
		30-39 Gy	50 (14.1)	62 (15.2)	35 (12.9)	60 (15.2)
		40-49 Gy	11 (3.1)	13 (3.2)	6 (2.2)	14 (3.5)
		50+ Gy	5 (1.4)	6 (1.5)	4 (1.5)	6 (1.5)
	Neck RT dose	None	143 (40.3)	153 (37.4)	116 (42.8)	149 (37.6)
		<10 Gy	121 (34.1)	147 (35.9)	92 (33.9)	138 (34.8)
		10-19 Gy	2 (0.6)	2 (0.5)	0 (0)	2 (0.5)
		20-29 Gy	20 (5.6)	24 (5.9)	16 (5.9)	23 (5.8)
		30-39 Gy	51 (14.4)	63 (15.4)	37 (13.7)	64 (16.2)
		40-49 Gy	17 (4.8)	18 (4.4)	9 (3.3)	18 (4.5)
		50+ Gy	1 (0.3)	2 (0.5)	1 (0.4)	2 (0.5)
	TBI dose	None	357 (100)	410 (100)	272 (100)	397 (100)
	Max Brain RT dose	None	143 (40.5)	153 (37.6)	116 (43.1)	149 (37.8)
		<10 Gy	12 (3.4)	12 (2.9)	9 (3.3)	13 (3.3)
		20-29 Gy	0 (0)	0 (0)	0 (0)	1 (0.3)
		30-39 Gy	5 (1.4)	4 (1.0)	3 (1.1)	5 (1.3)

© 2023 Phillips NS et al. *JAMA Network Open.*

			NCQ			
			Memory	Emotion	Task efficiency	Organization
Diagnosis group			N (%)	N (%)	N (%)	N (%)
		40-49 Gy	36 (10.2)	40 (9.8)	27 (10.0)	40 (10.2)
		50+ Gy	157 (44.5)	198 (48.6)	114 (42.4)	186 (47.2)
	Infratentorial RT dose	None	143 (40.6)	153 (37.7)	116 (43.3)	149 (37.9)
		<10 Gy	50 (14.2)	66 (16.3)	41 (15.3)	63 (16.0)
		10-19 Gy	1 (0.3)	1 (0.2)	0 (0)	0 (0)
		20-29 Gy	3 (0.9)	3 (0.7)	2 (0.7)	4 (1.0)
		30-39 Gy	12 (3.4)	13 (3.2)	9 (3.4)	14 (3.6)
		40-49 Gy	30 (8.5)	32 (7.9)	22 (8.2)	35 (8.9)
		50+ Gy	113 (32.1)	138 (34.0)	78 (29.1)	128 (32.6)
	Temporal lobe RT dose	None	143 (40.6)	153 (37.7)	116 (43.3)	149 (37.9)
		<10 Gy	39 (11.1)	46 (11.3)	33 (12.3)	44 (11.2)
		20-29 Gy	5 (1.4)	7 (1.7)	3 (1.1)	8 (2.0)
		30-39 Gy	22 (6.3)	26 (6.4)	19 (7.1)	28 (7.1)
		40-49 Gy	45 (12.8)	50 (12.3)	31 (11.6)	51 (13.0)
		50+ Gy	98 (27.8)	124 (30.5)	66 (24.6)	113 (28.8)

© 2023 Phillips NS et al. *JAMA Network Open.*

			NCQ			
			Memory	Emotion	Task efficiency	Organization
**Diagnosis group**			**N (%)**	**N (%)**	**N (%)**	**N (%)**
	Frontal lobe RT dose	None	143 (40.6)	153 (37.7)	116 (43.3)	149 (37.9)
		<10 Gy	97 (27.6)	112 (27.6)	75 (28.0)	108 (27.5)
		20-29 Gy	8 (2.3)	10 (2.5)	6 (2.2)	10 (2.5)
		30-39 Gy	41 (11.6)	54 (13.3)	29 (10.8)	48 (12.2)
		40-49 Gy	40 (11.4)	42 (10.3)	24 (9.0)	47 (12.0)
		50+ Gy	23 (6.5)	35 (8.6)	18 (6.7)	31 (7.9)
	Parietal/Occipital RT dose	None	143 (40.6)	153 (37.7)	116 (43.3)	149 (37.9)
		<10 Gy	87 (24.7)	104 (25.6)	70 (26.1)	103 (26.2)
		20-29 Gy	5 (1.4)	7 (1.7)	4 (1.5)	8 (2.0)
		30-39 Gy	33 (9.4)	44 (10.8)	25 (9.3)	41 (10.4)
		40-49 Gy	41 (11.6)	44 (10.8)	24 (9.0)	49 (12.5)
		50+ Gy	43 (12.2)	54 (13.3)	29 (10.8)	43 (10.9)
	CNS surgery including shunt	Yes	350 (94.9)	403 (95.3)	262 (93.2)	385 (94.8)
Hodgkin	Total		518 (100)	520 (100)	510 (100)	523 (100)
	Medical record abstraction	0	23 (4.4)	23 (4.4)	25 (4.9)	27 (5.2)

© 2023 Phillips NS et al. *JAMA Network Open.*

			NCQ			
			**Memory**	**Emotion**	**Task efficiency**	**Organization**
**Diagnosis group**			**N (%)**	**N (%)**	**N (%)**	**N (%)**
		Yes	495 (95.6)	497 (95.6)	485 (95.1)	496 (94.8)
	Any platinum drug	Yes	1 (0.2)	1 (0.2)	1 (0.2)	1 (0.2)
	Any anthracycline	Yes	93 (18.9)	99 (20.0)	99 (20.5)	96 (19.5)
	Any alkylator	Yes	270 (55.1)	271 (55.2)	265 (55.3)	277 (56.5)
	IV methotrexate	Yes	6 (1.2)	6 (1.2)	5 (1.0)	5 (1.0)
	Ara-C IV	Yes	3 (0.6)	3 (0.6)	3 (0.6)	3 (0.6)
	Any Ara-C	Yes	3 (0.6)	3 (0.6)	3 (0.6)	3 (0.6)
	Dexamethasone	Yes	11 (2.2)	11 (2.2)	11 (2.3)	9 (1.8)
	Prednisone	Yes	199 (41.0)	204 (41.7)	196 (41.2)	200 (41.2)
	IV Bleomycin	Yes	86 (17.5)	91 (18.4)	91 (18.9)	87 (17.6)
	SubQ Bleomycin	Yes	5 (1.0)	4 (0.8)	4 (0.8)	3 (0.6)
	Vincristine	Yes	219 (44.2)	222 (44.7)	219 (45.2)	226 (45.6)
	BCNU	Yes	5 (1.0)	6 (1.2)	6 (1.2)	5 (1.0)
	CCNU	Yes	29 (5.9)	28 (5.7)	28 (5.8)	25 (5.1)
	IV cyclophosphamide	Yes	81 (16.4)	82 (16.6)	86 (17.8)	87 (17.6)

© 2023 Phillips NS et al. *JAMA Network Open.*

			NCQ			
			**Memory**	**Emotion**	**Task efficiency**	**Organization**
**Diagnosis group**			**N (%)**	**N (%)**	**N (%)**	**N (%)**
	Chlorambucil	Yes	4 (0.8)	5 (1.0)	5 (1.0)	5 (1.0)
	Ifosfamide	Yes	1 (0.2)	1 (0.2)	1 (0.2)	0 (0)
	Nitrogen mustard	Yes	169 (34.5)	171 (34.8)	161 (33.5)	170 (34.6)
	Procarbazine	Yes	260 (53.2)	262 (53.5)	254 (53.1)	266 (54.4)
	Thiotepa	Yes	9 (1.8)	7 (1.4)	7 (1.4)	8 (1.6)
	DTIC	Yes	63 (12.9)	68 (13.8)	68 (14.2)	63 (12.9)
	Streptozocin	Yes	2 (0.4)	2 (0.4)	2 (0.4)	2 (0.4)
	Hexamethylmelamine	Yes	2 (0.4)	2 (0.4)	2 (0.4)	1 (0.2)
	Methotrexate route	IT+PO or PO only	487 (98.8)	489 (98.8)	478 (99.0)	489 (99.0)
		Other	6 (1.2)	6 (1.2)	5 (1.0)	5 (1.0)
	Any radiation	Yes	466 (93.2)	462 (92.4)	456 (93.1)	466 (93.0)
	Direct Brain RT	Yes	2 (0.4)	2 (0.4)	1 (0.2)	2 (0.4)
	Chest RT dose	Yes	419 (85.3)	414 (84.0)	409 (85.0)	422 (85.6)
	Direct Neck RT	Yes	431 (87.8)	429 (87.0)	423 (87.9)	435 (88.2)
	Direct abdomen RT	Yes	264 (53.8)	258 (52.3)	257 (53.4)	269 (54.6)

© 2023 Phillips NS et al. *JAMA Network Open.*

			NCQ			
			Memory	Emotion	Task efficiency	Organization
Diagnosis group			N (%)	N (%)	N (%)	N (%)
	Direct Pelvic RT	Yes	162 (33.0)	164 (33.3)	161 (33.5)	161 (32.7)
	Cumulative anthracycline dose (doxo-equivalent)	None	399 (81.9)	395 (80.6)	383 (80.3)	397 (81.4)
		<100 mg	6 (1.2)	6 (1.2)	6 (1.3)	6 (1.2)
		100-249 mg	49 (10.1)	53 (10.8)	53 (11.1)	49 (10.0)
		250+ mg	33 (6.8)	36 (7.3)	35 (7.3)	36 (7.4)
	Cumulative alkylator dose (cyclo-equivalent)	None	220 (52.0)	220 (51.8)	214 (51.7)	213 (50.5)
		<4000 mg/m2	10 (2.4)	8 (1.9)	9 (2.2)	10 (2.4)
		4000-7999 mg/m2	39 (9.2)	44 (10.4)	45 (10.9)	44 (10.4)
		8000+ mg/m2	154 (36.4)	153 (36.0)	146 (35.3)	155 (36.7)
	Cumulative platinum dose (cisplatin-equivalent)	None	492 (100)	494 (100)	482 (100)	493 (100)
	IV methotrexate dose	None	487 (98.8)	489 (98.8)	478 (99.0)	489 (99.0)
		<4300 mg/m2	6 (1.2)	6 (1.2)	5 (1.0)	5 (1.0)
	Chest RT dose	None	34 (6.9)	38 (7.7)	34 (7.1)	35 (7.1)

© 2023 Phillips NS et al. *JAMA Network Open.*

			NCQ			
			**Memory**	**Emotion**	**Task efficiency**	**Organization**
**Diagnosis group**			**N (%)**	**N (%)**	**N (%)**	**N (%)**
		<10 Gy	41 (8.4)	44 (8.9)	41 (8.5)	39 (7.9)
		10-19 Gy	12 (2.4)	13 (2.6)	13 (2.7)	11 (2.2)
		20-29 Gy	76 (15.5)	72 (14.6)	80 (16.7)	79 (16.1)
		30-39 Gy	146 (29.8)	145 (29.5)	142 (29.6)	147 (29.9)
		40-49 Gy	162 (33.1)	162 (32.9)	150 (31.3)	162 (32.9)
		50+ Gy	19 (3.9)	18 (3.7)	20 (4.2)	19 (3.9)
	Pelvic RT dose	None	34 (6.9)	38 (7.7)	34 (7.1)	35 (7.1)
		<10 Gy	296 (60.3)	292 (59.2)	287 (59.7)	298 (60.4)
		10-19 Gy	9 (1.8)	9 (1.8)	9 (1.9)	8 (1.6)
		20-29 Gy	32 (6.5)	31 (6.3)	35 (7.3)	32 (6.5)
		30-39 Gy	68 (13.8)	71 (14.4)	68 (14.1)	71 (14.4)
		40-49 Gy	52 (10.6)	52 (10.5)	48 (10.0)	49 (9.9)
	Abdomen RT dose	None	34 (6.9)	38 (7.7)	34 (7.1)	35 (7.1)
		<10 Gy	197 (40.1)	201 (40.8)	194 (40.3)	193 (39.1)
		10-19 Gy	12 (2.4)	12 (2.4)	12 (2.5)	12 (2.4)

© 2023 Phillips NS et al. *JAMA Network Open.*

			NCQ			
			Memory	Emotion	Task efficiency	Organization
Diagnosis group			N (%)	N (%)	N (%)	N (%)
		20-29 Gy	50 (10.2)	49 (9.9)	54 (11.2)	54 (11.0)
		30-39 Gy	127 (25.9)	120 (24.3)	120 (24.9)	127 (25.8)
		40-49 Gy	69 (14.1)	71 (14.4)	65 (13.5)	71 (14.4)
		50+ Gy	2 (0.4)	2 (0.4)	2 (0.4)	1 (0.2)
	Neck RT dose	None	34 (6.9)	38 (7.7)	34 (7.1)	35 (7.1)
		<10 Gy	27 (5.5)	27 (5.5)	25 (5.2)	24 (4.9)
		10-19 Gy	15 (3.1)	16 (3.3)	15 (3.1)	14 (2.8)
		20-29 Gy	89 (18.2)	86 (17.5)	93 (19.4)	92 (18.7)
		30-39 Gy	159 (32.4)	158 (32.1)	156 (32.5)	165 (33.5)
		40-49 Gy	155 (31.6)	157 (31.9)	146 (30.4)	152 (30.9)
		50+ Gy	11 (2.2)	10 (2.0)	11 (2.3)	10 (2.0)
	TBI dose	None	491 (100)	493 (100)	481 (100)	493 (100)
	Max Brain RT dose	None	34 (6.9)	38 (7.7)	34 (7.1)	35 (7.1)
		<10 Gy	455 (92.7)	453 (91.9)	446 (92.7)	456 (92.5)
		30-39 Gy	2 (0.4)	2 (0.4)	1 (0.2)	2 (0.4)

© 2023 Phillips NS et al. *JAMA Network Open.*

			NCQ			
			**Memory**	**Emotion**	**Task efficiency**	**Organization**
**Diagnosis group**			**N (%)**	**N (%)**	**N (%)**	**N (%)**
	Infratentorial RT dose	None	34 (6.9)	38 (7.7)	34 (7.1)	35 (7.1)
		<10 Gy	455 (92.7)	453 (91.9)	446 (92.7)	456 (92.5)
		30-39 Gy	2 (0.4)	2 (0.4)	1 (0.2)	2 (0.4)
	Temporal lobe RT dose	None	34 (6.9)	38 (7.7)	34 (7.1)	35 (7.1)
		<10 Gy	455 (92.7)	453 (91.9)	446 (92.7)	456 (92.5)
		30-39 Gy	2 (0.4)	2 (0.4)	1 (0.2)	2 (0.4)
	Frontal lobe RT dose	None	34 (6.9)	38 (7.7)	34 (7.1)	35 (7.1)
		<10 Gy	457 (93.1)	455 (92.3)	447 (92.9)	458 (92.9)
	Parietal/Occipital lobe RT dose	None	34 (6.9)	38 (7.7)	34 (7.1)	35 (7.1)
		<10 Gy	457 (93.1)	455 (92.3)	447 (92.9)	458 (92.9)
	CNS surgery including shunt	Yes	1 (0.2)	1 (0.2)	1 (0.2)	1 (0.2)

ALL: Acute Lymphoblastic Leukemia; Ara-C: Cytarabine; BCNU: Carmustine; CCNU: Lomustine; CNS: Central Nervous System; CRT: conformal radiation therapy; Gy: gray; IT: Intrathecal therapy; IT+IV: Intrathecal +Intravenous therapy; IT+IV+IM: Intrathecal+Intravenous Therapy+Intramuscular IT+IV+PO: Intrathecal +Intravenous Therapy +by mouth; IM: Intramuscular; IT+PO: Intrathecal + by mouth; IV: intravenous; <: less than; mg: milligram; mg/m2: milligram/square meter; NCQ: Neurocognitive Questionnaire; %: percent; +: plus; PNET: Primitive Neuro-Ectodermal Tumors; PO: by mouth; RT: rhabdoid tumor; SubQ: Subcutaneous

© 2023 Phillips NS et al. *JAMA Network Open.*

eTable 2. Incidence of Chronic Condition by Diagnosis Group

		CNS	HL	ALL no/CRT	ALL w/CRT
		**N (%)**	**N (%)**	**N (%)**	**N (%)**
	Total	488 (100)	571 (100)	455 (100)	861 (100)
Grade 3-4 endocrine condition	Yes	53 (10.9)	147 (25.7)	25 (5.5)	82 (9.5)
Thyroid nodules	Yes	17 (3.5)	103 (18.0)	4 (0.9)	23 (2.7)
Diabetes	Yes	3 (0.6)	5 (0.9)	10 (2.2)	25 (2.9)
Gonad dysfunction	Yes	30 (6.1)	51 (8.9)	11 (2.4)	40 (4.6)
Another endocrine	Yes	11 (2.3)	0 (0.0)	0 (0.0)	1 (0.1)
Grade 3-4 cardiopulmonary condition	Yes	37 (7.8)	89 (15.6)	20 (4.4)	58 (6.7)
Heart attack	Yes	3 (0.6)	36 (6.3)	3 (0.7)	9 (1.0)
Congestive heart failure	Yes	1 (0.2)	19 (3.3)	4 (0.9)	5 (0.6)
Leaky valve	Yes	1 (0.2)	6 (1.1	1 (0.2)	0 (0.0)
Pericardial	Yes	0 (0.0)	2 (0.4)	0 (0.0)	2 (0.2)
Blood clot	Yes	31 (6.4)	22 (3.9)	10 (2.2)	41 (4.8)
Other cardiac	Yes	0 (0.0)	2 (0.4)	0 (0.0)	0 (0.0)
Emphysema	Yes	2 (0.4)	5 (0.9)	1 (0.2)	0 (0.0)
Lung fibrosis	Yes	2 (0.4)	16 (2.8)	2 (0.4)	2 (0.2)
Other respiratory	Yes	1 (0.2)	4 (0.7)	1 (0.2)	2 (0.2)
[a]Grade 3-4 neurological condition	Yes	157 (32.2)	12 (2.1)	16 (3.5)	69 (8.0)
Stroke	Yes	31 (6.4)	5 (0.9)	5 (1.1)	22 (2.6)
Paralysis	Yes	47 (9.6)	5 (0.9)	7(1.5)	11 (1.3)
Balance problems	Yes	30 (6.1)	3 (0.5)	1 (0.2)	6 (0.7)
Other neurologic	Yes	21 (4.3)	0 (0.0)	3 (0.7)	2 (0.2)
Seizures/epilepsy	Yes	79 (16.2)	1 (0.2)	8 (1.8)	43 (5.0)

ALL: Acute Lymphoblastic Leukemia; CNS: Central Nervous System; CRT: conformal radiation therapy; HL: Hodgkins Lymphoma; %: percent

© 2023 Phillips NS et al. *JAMA Network Open.*

[a]Grading of neurological condition excluded memory problems since it is considered an outcome variable.

© 2023 Phillips NS et al. *JAMA Network Open.*

eTable 3. Prevalence and Relative Risk of Neurocognitive Impairment at Follow-up in Survivors Compared to Siblings Among Those With Unimpaired Function in the Domain at Baseline

		**Memory**	**Emotion**	**Task Efficiency**	**Organization**
**ALL no/Cranial Radiotherapy**	Prevalence (95%CI)	14.0 (10.7-17.4)	7.7 (5.1-10.3)	10.4 (7.4-13.5)	4.7 (2.6- 6.7)
	Relative risk (95%CI)	1.61 (0.93-2.81)	1.44 (0.66-3.14)	1.35 (0.73-2.49)	1.82 (0.71-4.71)
**ALL w/Cranial Radiotherapy**	Prevalence (95%CI)	25.8 (22.6-29.0)	11.2 (9.0-13.5)	15.7 (12.8-18.6)	6.1 (4.4- 7.7)
	Relative risk (95%CI)	**3.22 (1.98-5.24)**	**2.00 (1.08-3.70)**	**2.24 (1.26-4.00)**	2.22 (0.94-5.25)
**CNS**	Prevalence (95%CI)	34.7 (30.0-39.5)	15.5 (12.1-18.9)	22.8 (17.9-27.6)	14.5 (11.2-17.9)
	Relative risk (95%CI)	**4.23 (2.60-6.86)**	**2.82 (1.51-5.27)**	**2.93 (1.67-5.16)**	**4.85 (2.14-11.0)**
**HL**	Prevalence (95%CI)	16.6 (13.4-19.8)	6.9 (4.8- 9.1)	12.0 (9.2-14.8)	5.7 (3.8- 7.7)
	Relative risk (95%CI)	**2.02 (1.21-3.37)**	1.80 (0.87-3.71)	1.61 (0.90-2.86)	1.63 (0.69-3.82)
**Siblings**	Prevalence (95%CI)	7.8 (4.3-11.4)	5.0 (2.1- 7.9)	7.0 (3.5-10.5)	2.9 (0.6- 5.2)
	Relative risk (95%CI)	REF	REF	REF	REF

ALL: Acute Lymphoblastic Leukemia; CNS: Central Nervous System; CRT: Cranial Radiation Therapy; HL: Hodgkin Lymphoma,

Bold indicates significant RR at P<0.05

© 2023 Phillips NS et al. *JAMA Network Open.*

eTable 4. Univariate Models for Radiation Exposure by Dose in CNS Tumor Survivor and Risk for New-Onset Neurocognitive Impairment at Follow-up (T2) Among Those With No Impairment at Baseline (T1).

One model per treatment, adjusted for sex and age at T1. Referent is no radiation

		Memory		Emotion		Task efficiency		Organization	
**Covariate**	**Level**	**Relative risk (95%CI)**	**P-value**	**Relative risk (95%CI)**	**P-value**	**Relative risk (95%CI)**	**P-value**	**Relative risk (95%CI)**	**P-value**
Infratentorial dose	1- <40 Gy	**1.77 (1.00-3.13)**	**0.0500**	2.06 (0.89-4.77)	0.0929	1.49 (0.53-4.19)	0.4502	0.83 (0.21-3.24)	0.7891
	40- <50 Gy	1.30 (0.74-2.27)	0.3585	1.18 (0.49-2.82)	0.7074	1.35 (0.59-3.09)	0.4760	0.71 (0.23-2.24)	0.5610
	50+ Gy	**1.75 (1.27-2.41)**	**0.0006**	1.27 (0.76-2.13)	0.3571	1.59 (0.96-2.61)	0.0689	1.41 (0.84-2.36)	0.1967
Temporal lobe dose	1-<40 Gy	**1.70 (1.03-2.82)**	**0.0387**	1.64 (0.81-3.33)	0.1695	1.73 (0.86-3.46)	0.1237	1.08 (0.44-2.68)	0.8607
	40-<50 Gy	1.37 (0.83-2.25)	0.2220	1.13 (0.54-2.36)	0.7504	1.83 (0.94-3.58)	0.0754	1.31 (0.61-2.83)	0.4851
	50+ Gy	**2.05 (1.47-2.86)**	**<.0001**	1.14 (0.66-1.99)	0.6386	1.55 (0.89-2.71)	0.1213	1.41 (0.79-2.52)	0.2457
Frontal lobe dose	1-<40 Gy	1.40 (0.95-2.07)	0.0922	1.36 (0.74-2.50)	0.3200	1.48 (0.79-2.77)	0.2176	0.99 (0.46-2.16)	0.9811
	40-<50 Gy	**1.65 (1.12-2.43)**	**0.0116**	0.87 (0.36-2.13)	0.7667	1.78 (0.89-3.58)	0.1024	1.85 (0.96-3.55)	0.0645
	50+ Gy	1.52 (0.92-2.52)	0.1029	**2.26 (1.25-4.07)**	**0.0066**	**2.27 (1.18-4.34)**	**0.0135**	1.61 (0.75-3.47)	0.2242
Parietal/Occipital dose	1-<40 Gy	1.47 (0.96-2.27)	0.0780	1.09 (0.54-2.20)	0.8157	1.13 (0.51-2.47)	0.7651	1.14 (0.53-2.45)	0.7357
	40-<50 Gy	**1.71 (1.15-2.56)**	**0.0081**	1.28 (0.62-2.61)	0.5038	**2.09 (1.08-4.05)**	**0.0294**	1.75 (0.92-3.33)	0.0901

© 2023 Phillips NS et al. *JAMA Network Open.*

	50+ Gy	**1.85 (1.27-2.70)**	**0.0013**	1.46 (0.77-2.77)	0.2499	**2.10 (1.18-3.72)**	**0.0112**	0.99 (0.41-2.39)	0.9853

CI: confidence interval; Gy: gray; <: less than; %: percent; +: plus; P: probability

© 2023 Phillips NS et al. *JAMA Network Open.*

eTable 5. Multivariable Analysis of Timing of Chronic Condition Onset and Relative Risk for New-Onset Neurocognitive Impairment in Cognitive Function Domains Among Survivors With No Impairment in the Domain at Baseline

**Cognitive function domain**	**Chronic Conditions** (Grade 3-4 vs 0-2)	**Timing of conditions**	**Relative risk** (95%CI)
**ALL Survivors Treated with Cranial Radiation**			
Memory	Cardiopulmonary	New-onset	**1.74 (1.13-2.67)**
		Established	1.35 (0.88-2.06)
	Endocrine	New-onset	**1.64 (1.12-2.40)**
		Established	1.04 (0.69-1.58)
Task Efficiency	Cardiopulmonary	New-onset	1.37 (0.53-3.54)
		Established	**1.83 (1.02-3.27)**
Organization	Endocrine	New-onset	0.39(0.06-2.79)
		Established	2.01 (0.99-4.07)
**ALL Survivors Treated with Chemotherapy-only**			
Memory	Neurologic	New-onset	**3.68 (1.30-10.4)**
		Established	1.50 (0.56-4.01)
Emotion	Neurologic	New-onset	**9.14 (4.37-19.1)**
		Established	2.45 (0.63-9.50)
Task Efficiency	Cardiopulmonary	New-onset	1.48 (0.20-11)
		Established	**3.04(1.19-7.78)**
	Endocrine	New-onset	1.81 (0.50-6.51)
		Established	**3.10 (1.34-7.17)**
**CNS Tumor Survivors**			
Memory	Neurologic	New-onset	**2.32 (1.64-3.28)**
		Established	1.18 (0.87-1.60)
Emotion	Neurologic	New-onset	1.94 (0.88-4.28)
		Established	**1.80 (1.14-2.83)**

© 2023 Phillips NS et al. *JAMA Network Open.*

Task Efficiency	Neurologic	New-onset	**3.25 (1.55-6.81)**
		Established	**2.19 (1.42-3.38)**
Organization	Cardiopulmonary	New-onset	1.67 (0.67-4.12)
		Established	**2.07 (1.14-3.75)**
**Hodgkin Lymphoma Survivors**			
Memory	Cardiopulmonary	New-onset	**1.66 (1.06-2.60)**
		Established	1.38 (0.79-2.39)
	Neurologic	New-onset	**2.67 (1.64-4.35)**
		Established	1.46 (0.58-3.69)
Task Efficiency	Neurologic	New-onset	**3.27 (1.75-6.14)**
		Established	2.28 (0.72-7.26)

One model per diagnosis group and domain, adjusted for age and sex. Only models with significant results shown. New-onset chronic condition includes survivors who had grade 0-2 chronic conditions at baseline but developed a grade 3-4 chronic condition by follow-up. Established level includes survivors who had a grade 3-4 chronic condition at both baseline and follow-up. Cardiopulmonary condition excludes stroke and Neurologic condition include both stroke and seizure. Bold indicates significant RR. Reference group for chronic conditions is those individuals who had neither a new-onset nor an established grade 3-4 condition of the relevant type. Supplemental Table 2 describes the prevalence of specific diseases associated with each chronic condition category.

ALL: Acute Lymphoblastic Leukemia; CI: confidence interval; CNS: Central Nervous System

© 2023 Phillips NS et al. *JAMA Network Open.*

eTable 6: Effects of Treatment and Evaluating Mediation by Chronic Health Conditions on Late Onset of Cognitive Impairment Among Childhood Cancer Survivors

**Neurocognitive Outcome**	**Cancer Diagnosis[a]**	**Treatment[b]**	**Direct Effect of Treatment on Neurocognitive Outcome**			**CHC Mediator[c]**	**Reporting of CHC**	**Indirect Effect of Treatment on Neurocognitive Outcome Through CHC**			
			**Std Est**	**Std Error**	**p**			**Std Est**	**Std Error**	**p**	**Proportion mediated**
Memory	ALL with chemotherapy only	Alkylator <8000 mg/m^2	0.62	0.41	0.13	Neurologic	Established	0.11	0.98	0.91	0.15
							New-Onset	-8.53	8.89	0.34	0.06
		Alkylator >8000 mg/m^2	1.03	0.40	0.01	Neurologic	Established	-0.05	0.50	0.91	0.06
							New-Onset	1.69	1.21	0.16	0.05
		Ara-C	-0.76	0.38	0.05	Neurologic	Established	-0.02	0.18	0.92	0.02
							New-Onset	-1.33	1.10	0.23	0.02
	ALL with CRT	Methotrexate (IT + IV + IM)	0.14	0.22	0.51	Cardio-pulmonary	Established	0.33	0.26	0.20	0.75
							New-Onset	0.07	0.34	0.83	0.40
						Endocrine	Established	0.00	0.02	0.94	0.13
							New-Onset	0.35	0.31	0.27	0.13
		CRT dose >20 Gy	0.15	0.14	0.30	Cardio-pulmonary	Established	0.01	0.09	0.90	0.08
							New-Onset	-0.05	0.22	0.81	0.37
						Endocrine	Established	0.01	0.06	0.85	0.02
							New-Onset	-0.13	0.17	0.43	0.02
	CNS tumor	CSI with boost	**0.68**	**0.20**	**<0.001**	Neurologic	Established	-0.02	0.05	0.63	0.04

© 2023 Phillips NS et al. *JAMA Network Open.*

							**New-Onset**	**2.02**	**1.01**	**0.05**	**0.78**
		Focal radiation	**0.47**	**0.19**	**0.02**	Neurologic	Established	0.00	0.02	0.90	0.01
							New-Onset	1.75	0.96	0.07	0.82
		CRT dose	0.16	0.45	0.73	Neurologic	Established	0.01	0.05	0.78	0.21
							New-Onset	1.79	1.20	0.14	0.96
		CCNU	-0.06	0.23	0.80	Neurologic	Established	0.08	0.16	0.59	0.82
							**New-Onset**	**-9.59**	**3.00**	**<0.001**	**.99**
		VP shunt	0.00	0.16	0.98	Neurologic	Established	0.02	0.03	0.63	0.38
							New-Onset	-0.22	0.30	0.46	n-e
	Hodgkin lymphoma	Chest radiation <35 Gy	-0.28	0.47	0.55	Neurologic	Established	0.08	0.47	0.86	0.29
							New-Onset	-1.08	1.01	0.29	0.48
						Cardio-pulmonary	Established	0.68	0.65	0.29	0.29
							New-Onset	0.40	0.41	0.33	0.29
		Chest radiation >35 Gy	0.23	0.45	0.61	Neurologic	Established	0.02	0.28	0.96	0.04
							New-Onset	-1.48	1.05	0.16	0.82
						Cardio-pulmonary	Established	0.82	0.70	0.24	0.69
							New-Onset	0.72	0.48	0.14	0.69
		Any alkylator	0.10	0.23	0.68	Neurologic	Established	0.19	0.54	0.72	0.48
							New-Onset	**-2.27**	**0.95**	**0.02**	**0.99**
						Cardio-pulmonary	Established	-0.11	0.13	0.39	0.12
							New-Onset	0.11	0.12	0.38	0.12

© 2023 Phillips NS et al. *JAMA Network Open.*

		Any anthracycline	-0.28	0.46	0.54	Neurologic	Established	0.06	0.18	0.74	0.34
							New-Onset	0.57	0.18	0.72	0.17
						Cardio-pulmonary	Established	0.64	0.44	0.15	0.16
							New-Onset	-0.27	0.27	0.32	0.45
		Any bleomycin	0.37	0.40	0.35	Neurologic	Established	0.07	0.27	0.81	0.18
							New-Onset	1.73	1.41	0.22	0.44
						Cardio-pulmonary	Established	-0.49	0.36	0.18	0.05
							New-Onset	0.20	0.23	0.37	0.56
		Any CCNU	-0.04	0.43	0.92	Neurologic	Established	-3.61	8.95	0.69	0.46
							New-Onset	0.40	1.20	0.74	0.46
						Cardio-pulmonary	Established	0.05	0.20	0.79	0.75
							New-Onset	0.10	0.21	0.61	0.75
Emotional Regulation	ALL with chemotherapy only	Any Ara-C	-1.13	0.46	0.01	Neurologic	Established	0.03	0.48	0.95	0.03
							New-Onset	0.88	2.82	0.76	0.03
	CNS tumor	CSI with boost	0.25	0.32	0.43	Neurologic	Established	-0.10	0.15	0.50	0.41
							New-Onset	2.20	1.64	0.18	0.28
		Focal radiation	0.07	0.30	0.81	Neurologic	Established	0.11	0.13	0.41	0.99
							New-Onset	1.96	1.54	0.20	0.99
		CRT dose	0.83	0.48	0.08	Neurologic	Established	-0.10	0.32	0.75	0.15
							New-Onset	2.01	1.86	0.28	0.72
		Any CCNU	0.13	0.35	0.70	Neurologic	Established	**0.50**	**0.22**	**0.02**	**0.93**

© 2023 Phillips NS et al. *JAMA Network Open.*

						New-Onset	-9.90	6.31	0.12	0.72
		VP shunt	**-0.91**	**0.32**	**<0.001**	Neurologic	Established	0.16	0.12	0.19	0.21
							New-Onset	-0.34	0.32	0.30	0.27
Task Efficiency	ALL with chemotherapy only	Anthracycline >250 mg/m^2 or alkylator >8000 mg/m^2	**1.21**	**0.53**	**0.02**	Cardio-pulmonary	Established	1.79	0.97	0.06	0.73
						Endocrine	Established	1.71	0.92	0.06	0.73
		Anthracycline >250 mg/m^2 and alkylator >8000 mg/m^2	**0.94**	**0.40**	**0.02**	Cardio-pulmonary	Established	**2.12**	**1.03**	**0.04**	**0.68**
						Endocrine	Established	0.22	0.98	0.82	0.22
	ALL with CRT	Methotrexate (IT + IV + IM)	0.27	0.31	0.39	Cardio-pulmonary	Established	0.63	0.41	0.12	0.22
							New-Onset	-0.31	0.66	0.64	0.22
		CRT dose >20 Gy	0.36	0.22	0.09	Cardio-pulmonary	Established	-0.04	0.19	0.84	0.12
							New-Onset	-0.09	0.30	0.76	0.34
	CNS tumor	CSI with boost	**0.66**	**0.32**	**0.04**	Neurologic	Established	-0.18	0.24	0.45	0.37
							New-Onset	**15.96**	**5.98**	**0.01**	**0.96**
		Focal radiation	0.53	0.28	0.06	Neurologic	Established	-0.18	0.20	0.38	0.71
							New-Onset	**17.04**	**6.74**	**0.01**	**0.98**
		CRT dose	0.25	0.66	0.70	Neurologic	Established	-0.39	0.50	0.43	0.31
							New-Onset	**1.85**	**0.87**	**0.03**	**0.99**
		Any CCNU	-0.01	0.34	0.98	Neurologic	Established	**0.67**	**0.29**	**0.02**	**0.99**
							New-Onset	**-16.15**	**6.18**	**0.01**	**0.99**
		VP Shunt	-0.12	0.26	0.64	Neurologic	Established	0.10	0.17	0.55	0.44

© 2023 Phillips NS et al. *JAMA Network Open.*

							New-Onset	-0.24	0.68	0.72	0.58
	Hodgkin lymphoma	Chest radiation <35 Gy	0.12	0.52	0.82	Neurologic	Established	0.20	0.94	0.83	0.48
							New-Onset	-0.78	0.93	0.41	0.78
		Chest radiation >35Gy	0.00	0.53	1.00	Neurologic	Established	-0.82	0.73	0.26	n-e
							New-Onset	-1.10	0.97	0.26	n-e
		Any alkylator	**-0.55**	**0.28**	**0.05**	Neurologic	Established	-0.44	0.60	0.47	0.50
							New-Onset	-1.56	0.90	0.08	0.79
		Any anthracycline	0.09	0.46	0.84	Neurologic	Established	0.03	0.43	0.94	0.17
							New-Onset	-0.38	0.71	0.59	0.66
		Any bleomycin	0.74	0.41	0.07	Neurologic	Established	0.54	0.70	0.44	0.48
							New-Onset	1.59	0.85	0.06	0.73
		Any CCNU	0.07	0.53	0.89	Neurologic	Established	-8.50	7.12	0.23	0.11
							New-Onset	0.50	0.81	0.54	0.11
Organization	ALL with CRT	Methotrexate (IT + IV + IM)	-0.92	0.63	0.14	Endocrine	Established	-0.21	0.38	0.58	0.19
							New-Onset	-0.15	0.55	0.79	0.15
		CRT dose >20 Gy	0.48	0.32	0.13	Endocrine	Established	0.20	0.22	0.35	0.04
							New-Onset	-0.20	0.45	0.65	0.04
		IT Ara-C	**0.94**	**0.38**	**0.01**	Endocrine	Established	0.22	0.25	0.36	0.01
							New-Onset	-0.80	0.85	0.35	0.01
	CNS tumor	CSI with boost	0.19	0.33	0.57	Cardio-pulmonary	Established	-0.19	0.42	0.65	0.09
							New-Onset	0.60	0.51	0.24	0.09

© 2023 Phillips NS et al. *JAMA Network Open.*

		Focal radiation	-0.18	0.33	0.57	Cardio-pulmonary	Established	-0.67	0.38	0.08	0.14
							New-Onset	0.44	0.42	0.29	0.14
		Any CCNU	0.61	0.36	0.09	Cardio-pulmonary	Established	0.44	0.51	0.39	0.73
							New-Onset	-0.07	0.29	0.81	0.11
		VP shunt	0.02	0.28	0.93	Cardio-pulmonary	Established	0.01	0.36	0.98	0.14
							New-Onset	-0.24	0.33	0.46	0.82

ALL: acute lymphoblastic leukemia; Ara-C: cytarabine; CCNU: lomustine; CNS: central nervous system; CRT: cranial radiation therapy; CSI: craniospinal irradiation; Est: estimate; IM: intramuscular; IT: intrathecal; IV: intravenous; P: probability; Std: standard; VP: ventriculoperitoneal shun

Note: [a]All models by diagnosis group.
[b]Treatment models included sex, age, and all treatments listed within each diagnosis group.
[c]Separate models were generated for each mediator category.

© 2023 Phillips NS et al. *JAMA Network Open.*

eTable 7. Multivariable Analysis of Health Outcomes and Health Behaviors Associated With New-Onset Neurocognitive Impairment of the Measures in the CCSS-NCQ From Baseline to Follow-up Among Survivors With Unimpaired Neurocognitive Function in the Domain at Baseline

	**Neurocognitive Outcomes**			
	**Memory**	**Emotional Regulation**	**Task Efficiency**	**Organization**
	**ALL Survivors Treated with Cranial Radiation**			
	RR (95%CI)	RR (95%CI)	RR (95%CI)	RR (95%CI)
**Relapse/Subsequent Malignancy (Yes vs. No)**	**1.31 (1.00-1.73)**	0.76 (0.43-1.34)	1.02 (0.64-1.64)	0.72 (0.34-1.56)
**Anxiety (Yes vs. No)**	1.49 (0.93-2.39)	**2.92 (1.64-5.17)**	0.90 (0.31-2.63)	**3.30 (1.62 -6.71)**
**Depression (Yes vs. No)**	**1.92 (1.48 -2.50)**	**2.61 (1.68 -4.05)**	1.37 (0.85-2.19)	**3.47 (1.96-6.14)**
**Former Smoker (vs. Never)**	1.20 (0.81 -1.77)	1.55 (0.85 -2.81)	1.47 (0.85-2.53)	1.10 (0.48 -2.52)
**Current Smoker (vs. Never)**	**1.56 (1.11 -2.18)**	1.60 (0.90-2.82)	**2.04 (1.30-3.19)**	0.37 (0.09-1.56)
**BMI Obese (30 + vs. <25)**	0.91 (0.67-1.24)	1.32 (0.74-2.36)	1.27 (0.80-2.02)	1.07 (0.54-2.13)
**BMI Overweight (25-29 vs. <25)**	0.91 (0.67-1.22)	**1.79 (1.08-2.98)**	1.09 (0.68-1.75)	0.93 (0.47-1.85)
**Education <High school graduate (ref College graduate)**	1.63 (0.84-3.13)	**7.18 (3.88-13.3)**	**6.35 (3.86-10.4)**	2.01 (0.64-6.34)
**Education High school graduate (ref College graduate)**	**1.54 (1.10-2.16)**	**3.53 (2.07-6.00)**	**1.97 (1.12-3.48)**	0.67 (0.24-1.89)
**Education some college/vocational training (ref College graduate)**	1.19 (0.90-1.58)	1.61 (0.96-2.70)	**1.75 (1.14-2.71)**	1.13 (0.62-6.34)

© 2023 Phillips NS et al. *JAMA Network Open.*

**Meet CDC Exercise Guidelines (Yes vs. No)**	1.28 (0.99 -1.65)	1.01 (0.66-1.54)	1.26 (0.85-1.85)	**1.82 (1.00-3.30)**
**ALL Survivors Treated with Chemotherapy Only**				
	RR (95%CI)	RR (95%CI)	RR (95%CI)	RR (95%CI)
**Relapse/Subsequent Malignancy (Yes vs. No)**	0.90 (.35-2.32)	1.46 (0.53-4.04)	1.71 (0.68-4.30)	-
**Anxiety (Yes vs. No)**	1.29 (0.59-2.82)	2.30 (0.97-5.46)	**2.89 (1.39-6.01)**	2.53 (0.92-6.98)
**Depression (Yes vs. No)**	**1.68 (1.00-2.81)**	2.90 (1.45-5.80)	**2.40 (1.29-4.48)**	2.32 (0.96-5.56)
**Former Smoker (vs. Never)**	0.88 (0.43-1.79)	0.62 (0.19-2.03)	0.51 (0.19-1.38)	0.60 (0.13-2.62)
**Current Smoker (vs. Never)**	1.73 (0.98-3.04)	1.92 (0.89-4.16)	1.14 (0.50-2.60)	1.64 (0.57-4.73)
**BMI Obese (30 + vs. <25)**	1.20 (0.60-2.38)	0.88 (0.29-2.62)	1.45 (0.67-3.12)	1.39 (0.43-4.51)
**BMI Overweight (25-29 vs. <25)**	0.65 (0.33-1.26)	1.23 (0.58-2.62)	0.67 (0.31-1.46)	0.37 (0.09-1.62)
**Education <High school graduate (ref College graduate)**	**5.82 (3.20-10.6)**	**3.42 (1.03-11.4)**	**3.96 (1.08-14.5)**	3.39 (0.37-30.9)
**Education High school graduate (ref College graduate)**	1.44 (0.53-3.88)	1.63 (0.50-5.30)	0.49 (0.07-3.51)	3.20 (0.85-12.0)
**Education some college/vocational training (ref College graduate)**	1.28 (0.76-2.14)	0.98 (0.46-2.08)	1.55 (0.85-2.84)	1.55 (0.58-4.16)
**Meet CDC Exercise Guidelines (Yes vs. No)**	1.39 (0.85-2.27)	**4.27 (1.95-9.36)**	1.74 (0.94-3.23)	1.41 (0.58-3.43)
**CNS Tumor Survivors**				
	RR (95%CI)	RR (95%CI)	RR (95%CI)	RR (95%CI)
**Relapse/Subsequent Malignancy (Yes vs. No)**	1.29 (0.94-1.75)	0.87 (0.50-1.52)	1.21(0.73-2.01)	0.76 (0.40-1.43)
**Anxiety**	**1.93 (1.25-2.99)**	**3.00 (1.61-5.57)**	**2.17 (1.01-4.66)**	1.63 (0.77-3.46)

© 2023 Phillips NS et al. *JAMA Network Open.*

**(Yes vs. No)**				
**Depression (Yes vs. No)**	1.28 (0.88-1.86)	1.59 (0.93-2.71)	1.07 (0.56-2.05)	1.53 (0.87-2.68)
**Former Smoker (vs. Never)**	1.02 (0.66-1.57)	0.88 (0.39-1.95)	0.59 (0.25-1.39)	0.368 (0.12-1.18)
**Current Smoker (vs. Never)**	0.74 (0.41-1.32)	**2.34 (1.38-3.98)**	1.50 (0.86-2.60)	0.84 (0.35-2.00)
**BMI Obese (30 + vs. <25)**	1.26 (0.89-1.78)	0.81 (0.45-1.47)	1.46 (0.91-2.36)	1.02 (0.56-1.86)
**BMI Overweight (25-29 vs. <25)**	1.23 (0.88-1.72)	0.74 (0.42-1.28)	0.88 (0.50-1.55)	1.00 (0.82-3.24)
**Education <High school graduate (ref College graduate)**	1.77 (0.95-3.33)	1.65 (0.53-5.15)	**3.41 (1.69-6.91)**	1.12 (0.29-4.29)
**Education High school graduate (ref College graduate)**	**1.68 (1.17-2.42)**	**2.40 (1.32-4.34)**	1.72 (0.90-3.31)	1.78 (0.91-3.45)
**Education some college/vocational training (ref College grad)**	**1.42 (1.01-1.98)**	**1.83 (1.04-3.21)**	**1.67 (1.02-2.74)**	**1.81 (1.04-3.17)**
**Meet CDC Exercise Guidelines (Yes vs. No)**	**1.43 (1.09-1.89)**	**1.72 (1.10-2.69)**	1.46 (0.95-2.25)	**2.51 (1.53-4.10)**
**Hodgkin Lymphoma Survivors**				
	RR (95%CI)	RR (95%CI)	RR (95%CI)	RR (95%CI)
**Relapse/Subsequent Malignancy (Yes vs. No)**	1.33 (0.90-1.97)	0.96 (0.49-1.88)	1.32 (0.83-2.10)	1.22 (0.61-2.42)
**Anxiety (Yes vs. No)**	**2.59 (1.58-4.25)**	1.84 (0.59-5.73)	1.65 (0.75-3.63)	1.40 (0.47-4.20)
**Depression (Yes vs. No)**	1.52 (0.95-2.42)	1.01 (0.40-2.57)	**2.31 (1.38-3.85)**	1.18 (0.52-2.67)
**Former Smoker (vs. Never)**	1.09 (0.70-1.70)	1.69 (0.80-3.57)	1.00 (0.56-1.78)	0.95 (0.41-2.88)
**Current Smoker (vs. Never)**	1.40 (0.79-2.50)	**2.83 (1.26-6.35)**	1.67 (0.88-3.16)	0.85 (0.27-2.75)

© 2023 Phillips NS et al. *JAMA Network Open.*

**BMI Obese (30 + vs. <25)**	1.25 (0.72-2.18)	1.00 (0.41-2.40)	1.27 (0.66-2.44)	1.09 (0.41-2.88)
**BMI Overweight (25-29 vs. <25)**	**1.72 (1.11-2.65)**	0.78 (0.35-1.75)	1.50 (0.86-2.62)	1.62 (0.71-3.72)
**Education <High school (ref College graduate)**	1.40 (0.21-9.49)	2.00 (0.29-13.8)	2.57 (0.76-8.71)	-
**Education High school (ref College graduate)**	1.43 (0.79-2.59)	**2.44 (1.11-5.39)**	0.90 (0.38-2.13)	1.08 (0.34-3.47)
**Education some college/vocational training (ref College graduate)**	1.19 (0.78-1.84)	0.81 (0.35-1.87)	0.90 (0.51-1.59)	0.95 (0.43-2.11)
**Meet CDC Exercise Guidelines (No vs. Yes)**	1.34 (0.91-1.96)	1.13 (0.59-2.15)	1.11 (0.69-1.81)	1.04 (0.50-2.18)

CI: Confidence Interval; <: less than; vs.: versus; ref: reference; RR: regular rhythm; CNS: central nervous system; CDC: Centers for Disease Control
Bold indicates statistically significant (p<0.05); "-" denotes not estimable.

© 2023 Phillips NS et al. *JAMA Network Open.*

eTable 8. Comparison of Survivors With Completed Baseline NCQ, Unimpaired on at Least 1 Scale, and Still Alive as of the Follow-up Mailing to Those Who Competed Both Baseline and Follow-up NCQ Questionnaires, by Diagnosis

This summary includes demographic, psychosocial, and condition information at follow up. Survivors who completed both baseline and follow up surveys were more likely to be female, college educated, employed, living independently, less likely to smoke, and more likely to have at least one chronic health condition compared to those who only completed the baseline questionnaire.

			**baseline**		**baseline +follow up**		
**DX group**			**N**	**%**	**N**	**%**	**P-value***
**ALL no CRT**	Total		205	100	455	100	
	Sex	Female	98	47.8	270	59.3	0.0058
		Male	107	52.2	185	40.7	
	Race/ethnicity	White NH	183	89.3	411	90.3	0.67
		non-white	22	10.7	44	9.7	
	Age at diagnosis	0-4	125	61.0	269	59.1	.
		5-9	55	26.8	123	27.0	
		10-14	20	9.8	43	9.5	
		15-20	5	2.4	20	4.4	
	Age	18-29	164	80.0	307	67.5	.
		30-39	37	18.0	125	27.5	
		40+	4	2.0	23	5.1	
	Education	<=HS graduate	31	15.1	43	9.5	0.0002
		>=College	66	32.2	224	49.2	
		Some college	108	52.7	188	41.3	
	Household income	<$20,000	18	8.8	33	7.3	0.45
		$20,000-<$40,000	57	27.8	104	22.9	
		$40,000-<$60,000	33	16.1	87	19.1	
		$60,000+	76	37.1	178	39.1	
		Unknown	21	10.2	53	11.6	
	Personal income	<$20,000	102	49.8	233	51.2	0.14
		$20,000-<$40,000	66	32.2	120	26.4	

© 2023 Phillips NS et al. *JAMA Network Open.*

		$40,000-<$60,000	22	10.7	46	10.1	
		$60,000+	12	5.9	49	10.8	
		Unknown	3	1.5	7	1.5	
	Employment	Care-home	9	4.4	27	6.0	0.45
		Full-time	143	70.4	283	62.5	
		Look-work	6	3.0	16	3.5	
		Part-time	33	16.3	79	17.4	
		Retired	0	0.0	1	0.2	
		Student	10	4.9	39	8.6	
		Unable	2	1.0	8	1.8	
	Independent living	Yes	132	64.4	323	71.0	0.090
		No	73	35.6	132	29.0	
	Smoking status	Current smoker	50	24.4	57	12.5	0.0007
		Former smoker	30	14.6	79	17.4	
		Never smoked	125	61.0	319	70.1	
	BMI	Underweight (<18.5)	4	2.0	19	4.2	0.060
		Healthy (18.5-24)	99	48.3	239	52.5	
		Overweight (25-29)	55	26.8	121	26.6	
		Obese (30+)	45	22.0	65	14.3	
		Unknown	2	1.0	11	2.4	
	Met CDC activity criteria	Yes	138	69.0	290	64.7	0.29
	Any grade 3-4 chronic condition	Yes	26	12.7	80	17.6	0.12
	Grade 3-4 endocrine condition	Yes	6	2.9	25	5.5	0.15
	Grade 3-4 cardiac condition	Yes	8	3.9	16	3.5	0.81

© 2023 Phillips NS et al. *JAMA Network Open.*

	Grade 3-4 pulmonary condition	Yes	2	1.0	4	0.9	0.90
	Grade 3-4 neurological condition	Yes	1	0.5	13	2.9	0.051
	Grade 3-4 cardiopulmonary condition	Yes	9	4.4	20	4.4	0.99
	Adverse global mental health	Yes	21	10.8	43	9.7	0.67
	Symptoms of depression	Yes	25	12.9	50	11.3	0.57
	Symptoms of somatization	Yes	26	13.4	44	10.0	0.20
	Symptoms of anxiety	Yes	18	9.3	36	8.1	0.64
	Memory impairment	Yes	18	8.8	48	10.5	0.48
	Emotional impairment	Yes	24	11.8	46	10.1	0.53
	Task efficiency impairment	Yes	26	12.7	52	11.4	0.64
	Organization impairment	Yes	44	21.6	70	15.4	0.052
**ALL with CRT**	Total		428	100	861	100	
	Sex	Female	190	44.4	460	53.4	0.0023
		Male	238	55.6	401	46.6	
	Race/ethnicity	White NH	388	90.7	792	92.0	0.42
		non-white	40	9.3	69	8.0	
	Age at diagnosis	0-4	236	55.1	385	44.7	.
		5-9	116	27.1	241	28.0	
		10-14	54	12.6	162	18.8	

© 2023 Phillips NS et al. *JAMA Network Open.*

		15-20	22	5.1	73	8.5	
	Age	18-29	231	54.0	390	45.3	.
		30-39	181	42.3	396	46.0	
		40+	16	3.7	75	8.7	
	Education	<=HS graduate	98	22.9	146	17.0	0.0008
		>=College	158	36.9	409	47.5	
		Some college	172	40.2	306	35.5	
	Household income	<$20,000	57	13.3	102	11.8	0.027
		$20,000-<$40,000	111	25.9	185	21.5	
		$40,000-<$60,000	72	16.8	172	20.0	
		$60,000+	106	24.8	277	32.2	
		Unknown	82	19.2	125	14.5	
	Personal income	<$20,000	183	42.8	405	47.0	0.18
		$20,000-<$40,000	152	35.5	260	30.2	
		$40,000-<$60,000	43	10.0	102	11.8	
		$60,000+	26	6.1	61	7.1	
		Unknown	24	5.6	33	3.8	
	Employment	Care-home	17	4.0	41	4.8	0.25
		Full-time	290	68.2	574	67.5	
		Look-work	32	7.5	50	5.9	
		Part-time	39	9.2	111	13.0	
		Student	19	4.5	33	3.9	
		Unable	25	5.9	38	4.5	
		Unemployed	3	0.7	4	0.5	
	Independent living	Yes	269	62.9	583	67.7	0.083
		No	159	37.1	278	32.3	
	Smoking status	Current smoker	64	15.0	96	11.1	0.082
		Former smoker	50	11.7	90	10.5	
		Never smoked	310	72.4	674	78.3	
		Unknown	4	0.9	1	0.1	

© 2023 Phillips NS et al. *JAMA Network Open.*

	BMI	Underweight (<18.5)	10	2.3	18	2.1	0.99
		Healthy (18.5-24)	137	32.0	276	32.1	
		Overweight (25-29)	132	30.8	270	31.4	
		Obese (30+)	122	28.5	253	29.4	
		Unknown	27	6.3	44	5.1	
	Met CDC activity criteria	Yes	232	57.1	480	57.3	0.95
	Any grade 3-4 chronic condition	Yes	78	18.2	240	27.9	0.0002
	Grade 3-4 endocrine condition	Yes	29	6.8	82	9.5	0.098
	Grade 3-4 cardiac condition	Yes	14	3.3	54	6.3	0.023
	Grade 3-4 pulmonary condition	Yes	2	0.5	4	0.5	0.99
	Grade 3-4 neurological condition	Yes	12	2.8	37	4.3	0.19
	Grade 3-4 cardiopulmonary condition	Yes	15	3.5	58	6.7	0.018
	Adverse global mental health	Yes	28	7.0	68	8.3	0.43
	Symptoms of depression	Yes	45	11.3	92	11.2	0.99
	Symptoms of somatization	Yes	49	12.3	96	11.7	0.78
	Symptoms of anxiety	Yes	22	5.5	46	5.6	0.94

© 2023 Phillips NS et al. *JAMA Network Open.*

	Memory impairment	Yes	65	15.2	135	15.7	0.81
	Emotional impairment	Yes	46	10.7	84	9.8	0.58
	Task efficiency impairment	Yes	51	11.9	103	12.0	0.98
	Organization impairment	Yes	124	29.0	254	29.6	0.83
**CNS**	Total		257	100	488	100	
	Sex	Female	114	44.4	255	52.3	0.041
		Male	143	55.6	233	47.7	
	Diagnosis	Astrocytoma	160	62.3	297	60.9	0.76
		Medulloblastoma, PNET	51	19.8	108	22.1	
		Other CNS tumors	46	17.9	83	17.0	
	Race/ethnicity	White NH	231	89.9	462	94.7	0.015
		non-white	26	10.1	26	5.3	
	Age at diagnosis	0-4	87	33.9	155	31.8	.
		5-9	83	32.3	143	29.3	
		10-14	55	21.4	140	28.7	
		15-20	32	12.5	50	10.2	
	Age	18-29	119	46.3	219	44.9	.
		30-39	111	43.2	209	42.8	
		40+	27	10.5	60	12.3	
	Education	<=HS graduate	84	32.7	106	21.7	<.0001
		>=College	72	28.0	221	45.3	
		Some college	99	38.5	161	33.0	
		Unknown	2	0.8	0	0.0	
	Household income	<$20,000	46	17.9	83	17.0	0.016
		$20,000-<$40,000	57	22.2	101	20.7	
		$40,000-<$60,000	43	16.7	73	15.0	
		$60,000+	46	17.9	152	31.1	
		Unknown	65	25.3	79	16.2	

© 2023 Phillips NS et al. *JAMA Network Open.*

	Personal income	<$20,000	147	57.2	286	58.6	0.24
		$20,000-<$40,000	62	24.1	108	22.1	
		$40,000-<$60,000	13	5.1	46	9.4	
		$60,000+	14	5.4	26	5.3	
		Unknown	21	8.2	22	4.5	
	Employment	Care-home	17	6.8	29	6.1	0.0072
		Full-time	113	45.0	241	50.4	
		Look-work	23	9.2	31	6.5	
		Part-time	35	13.9	89	18.6	
		Retired	0	0.0	1	0.2	
		Student	10	4.0	32	6.7	
		Unable	50	19.9	51	10.7	
		Unemployed	3	1.2	4	0.8	
	Independent living	Yes	139	54.1	292	59.8	0.13
		No	118	45.9	196	40.2	
	Smoking status	Current smoker	41	16.0	47	9.6	0.019
		Former smoker	34	13.2	55	11.3	
		Never smoked	181	70.4	386	79.1	
		Unknown	1	0.4	0	0.0	
	BMI	Underweight (<18.5)	6	2.3	17	3.5	0.094
		Healthy (18.5-24)	86	33.5	197	40.4	
		Overweight (25-29)	73	28.4	146	29.9	
		Obese (30+)	71	27.6	101	20.7	
		Unknown	21	8.2	27	5.5	
	Met CDC activity criteria	Yes	122	49.6	270	56.8	0.064
	Any grade 3-4 chronic condition	Yes	113	44.0	253	51.8	0.041
	Grade 3-4 endocrine condition	Yes	21	8.2	53	10.9	0.24

© 2023 Phillips NS et al. *JAMA Network Open.*

	Grade 3-4 cardiac condition	Yes	11	4.3	34	7.0	0.14
	Grade 3-4 pulmonary condition	Yes	0	0.0	4	0.8	0.15
	Grade 3-4 neurological condition	Yes	38	14.8	103	21.1	0.036
	Grade 3-4 cardiopulmonary condition	Yes	11	4.3	37	7.6	0.081
	Adverse global mental health	Yes	18	8.5	43	10.2	0.50
	Symptoms of depression	Yes	27	12.7	63	14.9	0.46
	Symptoms of somatization	Yes	20	9.4	50	11.8	0.37
	Symptoms of anxiety	Yes	18	8.5	30	7.1	0.53
	Memory impairment	Yes	57	22.3	101	20.7	0.63
	Emotional impairment	Yes	35	13.7	67	13.7	0.98
	Task efficiency impairment	Yes	31	12.1	49	10.0	0.39
	Organization impairment	Yes	119	46.5	197	40.5	0.11
**Hodgkin Lymphoma**	Total		209	100	571	100	
	Sex	Female	86	41.1	313	54.8	0.0007
		Male	123	58.9	258	45.2	
	Race/ethnicity	White NH	189	90.4	536	93.9	0.097
		non-white	20	9.6	35	6.1	

© 2023 Phillips NS et al. *JAMA Network Open.*

	Age at diagnosis	0-4	3	1.4	9	1.6	.
		5-9	46	22.0	59	10.3	
		10-14	71	34.0	216	37.8	
		15-20	89	42.6	287	50.3	
	Age	18-29	18	8.6	34	6.0	.
		30-39	117	56.0	293	51.3	
		40+	74	35.4	244	42.7	
	Education	<=HS graduate	36	17.2	61	10.7	0.011
		>=College	109	52.2	358	62.7	
		Some college	64	30.6	152	26.6	
	Household income	<$20,000	11	5.3	25	4.4	0.15
		$20,000-<$40,000	33	15.8	84	14.7	
		$40,000-<$60,000	47	22.5	97	17.0	
		$60,000+	107	51.2	347	60.8	
		Unknown	11	5.3	18	3.2	
	Personal income	<$20,000	56	26.8	133	23.3	0.66
		$20,000-<$40,000	60	28.7	159	27.8	
		$40,000-<$60,000	38	18.2	105	18.4	
		$60,000+	50	23.9	158	27.7	
		Unknown	5	2.4	16	2.8	
	Employment	Care-home	15	7.2	47	8.3	0.0035
		Full-time	154	74.4	436	76.9	
		Look-work	9	4.3	9	1.6	
		Part-time	15	7.2	60	10.6	
		Retired	1	0.5	2	0.4	
		Student	2	1.0	7	1.2	
		Unable	10	4.8	5	0.9	
		Unemployed	1	0.5	1	0.2	
	Independent living	Yes	188	90.0	541	94.7	0.017
		No	21	10.0	30	5.3	

© 2023 Phillips NS et al. *JAMA Network Open.*

	Smoking status	Current smoker	35	16.7	64	11.2	0.12
		Former smoker	48	23.0	137	24.0	
		Never smoked	126	60.3	370	64.8	
	BMI	Underweight (<18.5)	8	3.8	14	2.5	0.31
		Healthy (18.5-24)	86	41.1	276	48.3	
		Overweight (25-29)	74	35.4	178	31.2	
		Obese (30+)	33	15.8	93	16.3	
		Unknown	8	3.8	10	1.8	
	Met CDC activity criteria	Yes	125	60.7	359	63.9	0.42
	Any grade 3-4 chronic condition	Yes	70	33.5	272	47.6	0.0004
	Grade 3-4 endocrine condition	Yes	35	16.7	147	25.7	0.0085
	Grade 3-4 cardiac condition	Yes	25	12.0	79	13.8	0.50
	Grade 3-4 pulmonary condition	Yes	2	1.0	22	3.9	0.038
	Grade 3-4 neurological condition	Yes	6	2.9	12	2.1	0.53
	Grade 3-4 cardiopulmonary condition	Yes	27	12.9	89	15.6	0.35
	Adverse global mental health	Yes	16	7.8	27	4.8	0.11
	Symptoms of depression	Yes	19	9.3	28	5.0	0.027
	Symptoms of somatization	Yes	32	15.6	72	12.7	0.30

© 2023 Phillips NS et al. *JAMA Network Open.*

	Symptoms of anxiety	Yes	15	7.3	28	5.0	0.21
	Memory impairment	Yes	20	9.6	53	9.3	0.89
	Emotional impairment	Yes	25	12.0	47	8.2	0.12
	Task efficiency impairment	Yes	20	9.6	51	8.9	0.78
	Organization impairment	Yes	32	15.5	61	10.7	0.070

ALL: Acute Lymphoblastic Leukemia; BMI: body mass index; CDC: Centers for Disease Control; CNS: Central Nervous System; CRT: conformal radiation therapy; DX: diagnosis; $: dollar; =: equal; HS: high school; <: less than; >: more than; N: number; NH: non-Hispanic; %: percent; +: plus; PNET: Primitive Neuro-Ectodermal Tumors; P: probability

*P-value from chi-square test comparing variable distribution between those with only baseline NCQ versus those with baseline and follow up NCQ responses

© 2023 Phillips NS et al. *JAMA Network Open.*
