# Supplementary material for: Late-onset Cognitive Impairment and Modifiable Risk Factors in Adult Childhood Cancer Survivors
Source: JAMA Netw Open. 2023 May 31;6(5):e2316077. doi: 10.1001/jamanetworkopen.2023.16077 (PMC10233416; doi:10.1001/jamanetworkopen.2023.16077)
Supplement: Supplement 2. — Data Sharing Statement [file jamanetwopen-e2316077-s002.pdf]

Data Sharing Statement

Phillips. Late-onset Cognitive Impairment and Modifiable Risk Factors in Adult Childhood Cancer Survivors. *JAMA Netw Open*. Published May 31, 2023.
doi:10.1001/jamanetworkopen.2023.16077

Data
Data available: Yes
Data types: Deidentified participant data, Data dictionary
How to access data: All data collected for this study, including survivors' demographic and treatment information, questionnaires, and a data dictionary defining each variable, are available to researchers via the CCSS website (CCSS.stjude.org/public-access-data).
When available: With publication

Supporting Documents
Document types: None

Additional Information
Who can access the data: Researchers whose proposed use of the data has been approved. See https://ccss.stjude.org/ for details or contact ccss@stjude.org.
Types of analyses: Analysis for research purposes approved by the CCSS publication committee.
Mechanisms of data availability: Investigators interested in accessing these data for research purposes can submit an application of intent to the CCSS publication committee.
